# *p*-*tert*-Butylthiacalix[4]arenes functionalized by *N*-(4’-nitrophenyl)acetamide and *N*,*N*-diethylacetamide fragments: synthesis and binding of anionic guests

**DOI:** 10.3762/bjoc.13.188

**Published:** 2017-09-13

**Authors:** Alena A Vavilova, Ivan I Stoikov

**Affiliations:** 1 Kazan Federal University, Kremlevskaya, 18, Kazan 420008, Russian Federation

**Keywords:** anion binding, synthesis, thiacalixarenes, UV spectroscopy

## Abstract

New *p*-*tert*-butylthiacalix[4]arenes, which are mono-, 1,2-di- and tetrasubstituted at the lower rim containing *N*-(4’-nitrophenyl)acetamide and *N*,*N*-diethylacetamide groups in *cone* and *partial cone* conformations have been synthesized. Their complexation ability towards a number of tetrabutylammonium salts *n*-Bu_4_NX (X = F^−^, Cl^−^, Br^−^, I^−^, CH_3_CO_2_^−^, H_2_PO_4_^−^, NO_3_^−^) was studied by UV spectroscopy. The effective receptor for the anions studied as well as selective receptors for F^−^, CH_3_CO_2_^−^ and H_2_PO_4_^−^ ions, which based on the synthesized thiacalix[4]arenes, have been obtained. It was shown that *p*-*tert*-butylthiacalix[4]arene tetrasubstituted at the lower rim by *N*-(4’-nitrophenyl)acetamide moieties bonded to the anions studied with association constants within the range of 3.55 × 10^3^–7.94 × 10^5^ M^−1^. Besides, the binding selectivity for F^−^, Cl^−^, CH_3_CO_2_^−^, and H_2_PO_4_^−^ anions against other anions was in the range of 4.1–223.9. Substituting one or two fragments in the macrocycle with *N*,*N*-diethylacetamide groups significantly reduces the complexation ability of the receptor. In contrast to the 1,3-disubstituted macrocycle containing two *N*-(4’-nitrophenyl)acetamide moieties, the 1,2-disubstituted thiacalix[4]arene, which contains only one such fragment and a *N*,*N*-diethylacetamide moiety, selectively binds F^−^ anions.

## Introduction

Anions play a key role in many biochemical processes [1] as substrates and/or cofactors in enzymatic reactions [2], in the environment (phosphate and nitrate in the ponds provoking their eutrophication) [2–4], and in phase-transfer catalysis [5–6]. The dysfunction in the regulation of anions is a cause of many diseases [7–12]. Thus, selective synthetic receptors can be used in medicine as medicinal and diagnostic agents. However, the design and synthesis of the systems for recognizing anions remains one of the important scientific challenges in organic chemistry [13–23]. Anion-receptor biomimetics aimed at developing synthetic analogs of the natural compounds that offer a deeper understanding of a number of biological processes.

The design of anion receptors is quite a challenge for several reasons. The anions are larger in size than cations and therefore have a smaller charge in relation to their radius. It means that the electrostatic interactions for anions binding are weaker than for cations. Anions have various shape and geometry, and the design of receptors that are complementary to a specific type of anion is needed. (Thia)calix[4]arene derivatives are a favorable platform for the design of such structures [24–34]. Due to their macrocyclic nature and the possibility to modify them in three different ways (upper and lower rims and bridge fragments), they show the ability to selectively recognize and bind different types of substrates [35–41].

Previously, our scientific group showed that 2-bromo-*N*-(4’-nitrophenyl)acetamide is the regioselective alkylating reagent for *p*-*tert*-butylthiacalix[4]arene. In these reactions, derivatives of thiacalix[4]arene variously substituted at the lower rim form 1,2-, 1,3-di- and trisubstituted macrocycles [42], depending on the nature of the alkali metal carbonates and solvent. In this paper, we describe the regioselective synthesis of *p*-*tert*-butylthiacalix[4]arene monosubstituted at the lower rim by *N*,*N*-diethylacetamide fragment and its further functionalization with the *N*-(4’-nitrophenyl)acetamide moiety. We also calculated the proposed model of anion binding for the new and previously synthesized thiacalix[4]arenes and compared their complexation properties toward number of singly charged anions (F^−^, Cl^−^, Br^−^, I^−^, CH_3_CO_2_^−^, H_2_PO_4_^−^, NO_3_^−^). The obtained novel synthetic anion receptors hold promise for their potential application in the development of more sophisticated biomimetic materials and diagnostic agents.

## Results and Discussion

### Synthesis of *p*-*tert*-butylthiacalix[4]arene derivatives containing *N*-(4’-nitrophenyl)acetamide and *N*,*N*-diethylacetamide moieties at the lower rim

**Scheme 1:**
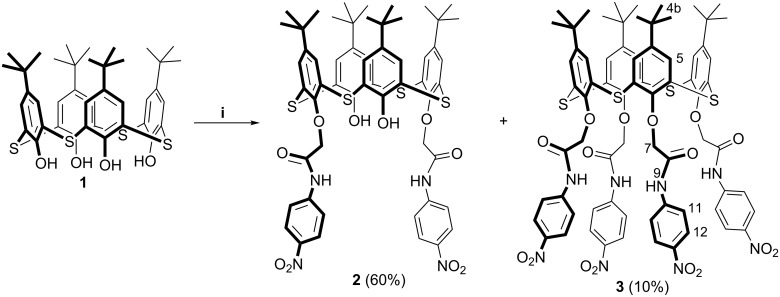
Synthesis of 1,3-di- and tetrasubstituted thiacalix[4]arenes **2** and **3**. Conditions: (i) macrocycle **1** (1 equiv), 2-bromo-*N*-(4’-nitrophenyl)acetamide (4 equiv), Na_2_CO_3_ (4 equiv), CH_3_CN, reflux.

The regioselective synthesis of *p*-*tert*-butylthiacalix[4]arenes partially substituted at the lower rim, is an important challenge because it allows the sequential functionalization of the macrocyclic platform with the necessary substituents. It was shown in acetonitrile using the weak base Na_2_CO_3_ that *p*-*tert*-butylthiacalix[4]arene **2**, 1,3-disubstituted at the lower rim, is formed with a low yield of 50% [42]. We estimated the effect of various reagent ratios for increasing the yield of the major product. The increase of the ratio to 1:4:4 = macrocycle **1**/Na_2_CO_3_/2-bromo-*N*-(4’-nitrophenyl)acetamide resulted in an increase of the yield of the 1,3-disubstituted thiacalix[4]arene to 60%. In addition, *p*-*tert*-butylthiacalix[4]arene **3** tetrasubstituted at the lower rim by *N*-(4’-nitrophenyl)acetamide fragments in *cone* conformation was obtained with 10% yield (Scheme 1). ^1^Н, ^13^С, 2D NOESY NMR, IR spectroscopy, mass spectrometry (MALDI–TOF) confirmed the macrocycle **3** structure. The elemental analysis gives us the composition of **3**.

In contrast to the ^1^H NMR spectrum of 1,3-disubstituted thiacalix[4]arene **2** [42], the signals of *tert*-butyl, oxymethylene and aryl protons are observed in the ^1^H NMR spectrum of tetrasubstituted macrocycle **3** as a single singlet. There are no signals of the hydroxy protons and only one singlet corresponding to amide protons is observed (see Supporting Information File 1, Figure S1). This indicates complete substitution of the initial macrocycle **1**.

In the MALDI–TOF mass spectrum of the compound **3** (M(C_72_H_72_N_8_O_16_S_4_) = 1432.4), a molecular ion peak with Na^+^ cation (*m*/*z* (M + Na^+^) = 1455.5) was observed (see Supporting Information File 1, Figure S4).

*p*-*tert*-Butylthiacalix[4]arenes monosubstituted at the lower rim are also valuable precursors for a further functionalization of the macrocycle. However, their preparation is significantly more difficult than the synthesis of 1,3-disubstituted thiacalix[4]arenes. Bulky functional groups able to form hydrogen bonds with free phenolic hydroxy groups and therefore block three residual hydroxy groups are usually applied in the synthesis of the monosubstituted derivatives of *p*-*tert*-butylthiacalix[4]arene [43]. For the synthesis of the monosubstituted derivative, the bulky *N*,*N*-diethylacetamide group able to participate in the formation of hydrogen bonds was used.

**Scheme 2:**
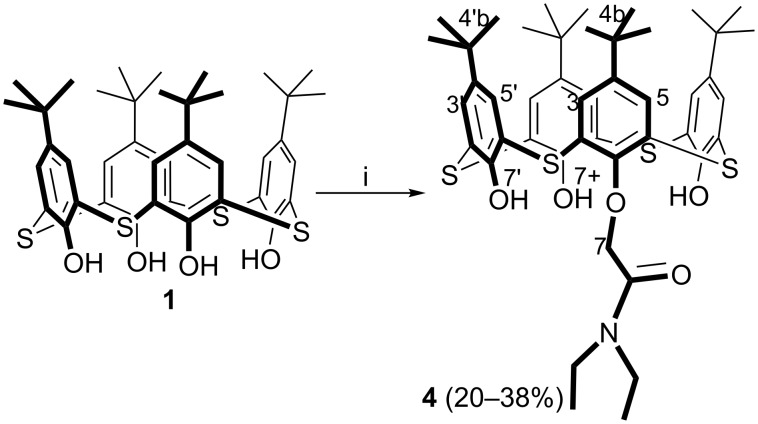
Synthesis of monosubstituted thiacalix[4]arene **4**. Conditions: (i) macrocycle **1** (1 equiv), 2-chloro-*N*,*N*-diethylacetamide (8 or 2 equiv), K_2_CO_3_ (26 equiv) or Cs_2_CO_3_ (4 equiv), acetone, reflux.

The monosubstituted derivative **4** was obtained by the alkylation of *p*-*tert*-butylthiacalix[4]arene (**1**) with 2-chloro-*N*,*N*-diethylacetamide in the presence of K_2_CO_3_ or Cs_2_CO_3_ (Scheme 2). Using ratio of *p*-*tert*-butylthiacalix[4]arene (**1**): alkylating reagent: Cs_2_CO_3_ = 1:2:4, the product **4** was isolated with 20% yield. With K_2_CO_3_ (ratio of *p*-*tert*-butylthiacalix[4]arene (**1**)**/**alkylating reagent/base = 1:8:26) the yield of **4** was 38%. According to ^1^H NMR spectrum, the remainder represents a mixture of the initial macrocycle and products of partial substitution at the lower rim. Apparently, the decrease in the yield of macrocycle **4** in the case of Cs_2_CO_3_ can be associated with the higher reactivity of the cesium phenolate formed in the reaction mixture, which facilitates further alkylation of *p*-*tert*-butylthiacalix[4]arene **4**.

The introduction of the *N*-(4’-nitrophenyl)acetamide group into the lower rim of *p*-*tert*-butylthiacalix[4]arene **4** is also of interest because this fragment contains a polar NH group able to interact with anionic substrates and a chromophore fragment necessary for the spectrophotometric detection of the complex formation. In this regard, the alkylation of monosubstituted derivative **4** with 2-bromo-*N*-(4’-nitrophenyl)acetamide was further studied in the presence of Na_2_CO_3_, K_2_CO_3_ and Cs_2_CO_3_.

**Scheme 3:**
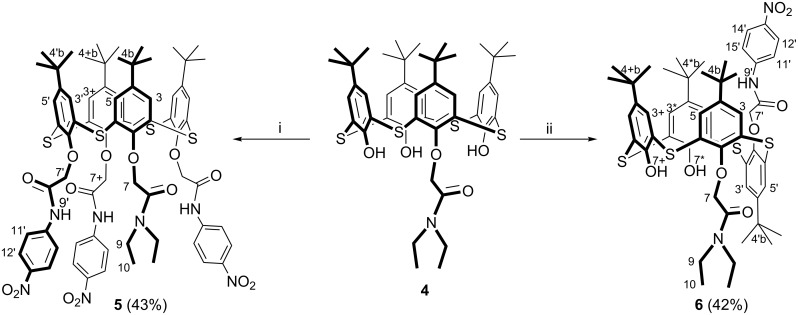
Synthesis of tetra- and 1,2-disubstituted thiacalix[4]arenes **5** and **6**, respectively. Conditions: (i) macrocycle **4** (1 equiv), 2-bromo-*N*-(4’-nitrophenyl)acetamide (6 equiv), Na_2_CO_3_ (6 equiv), acetone, reflux; (ii) macrocycle **4** (1 equiv), 2-bromo-*N*-(4’-nitrophenyl)acetamide (6 equiv), Cs_2_CO_3_ (6 equiv), acetone, reflux.

It was found that tetrasubstituted *p*-*tert*-butylthiacalix[4]arene **5** is formed with 43% yield in the presence of Na_2_CO_3_. Using Cs_2_CO_3_, the 1,2-disubstituted thiacalix[4]arene **6** was isolated as *partial cone* with 42% yield (Scheme 3). In the case of K_2_CO_3_, a hardly separable mixture of differently substituted derivatives formed.

The presence of two singlets (with an integral intensities of 3:1) of proton signals of *t*-Bu groups, one signal of oxymethylene protons and two singlets of OH groups in the ^1^H NMR spectrum of **4** confirms the formation of the monosubstituted product in *cone* conformation (see Supporting Information File 1, Figure S5). The absence of signals of OH groups in the ^1^H NMR spectrum of **5** indicates the complete substitution of the lower rim of initial macrocycle **4** (see Supporting Information File 1, Figure S9). Four singlets (with equal integrated intensities) of *t*-Bu groups in the ^1^H NMR spectrum show the asymmetric structure of **6** (see Supporting Information File 1, Figure S13). Also the presence of two singlets of OH groups and one singlet of NH group in the ^1^H NMR spectrum of **6** confirms the formation of disubstituted product.

The spatial structure of the compounds **4**–**6** was studied by ^1^H-^1^H NOESY NMR spectroscopy. In the NOESY NMR spectrum of the compound **4** (see Supporting Information File 1, Figure S7), the presence of cross peaks due to the dipole–dipole interaction of protons of the oxymethylene group with the hydroxy protons, as well as the cross peaks between the aromatic protons of the macrocycle, confirm that the macrocycle **4** is in the *cone* conformation. The cross peaks between the protons of substituents and the oxymethylene groups, the cross peaks between the protons of the amide groups and the *N*,*N*-diethylacetamide group, and also those between the aromatic protons of the macrocycle in the 2D NOESY NMR spectrum of **5** (see Supporting Information File 1, Figure S11) indicate that the macrocycle **5** is in the *cone* conformation. The presence of cross-peaks between the amide proton and protons of *tert*-butyl groups, the aromatic protons of the *p*-nitrophenyl substituent and protons of *tert*-butyl groups in 2D NOESY NMR spectrum of 1,2-disubstituted compound **6** (see Supporting Information File 1, Figure S15), as well as the cross peaks between the aromatic protons of the macrocycle and the oxymethylene protons of *N*,*N*-diethylacetamide moiety indicate the presence of substituents on opposite sides of the macrocyclic ring. This fact, as well as the cross-peak due to the dipole–dipole interaction of protons of the hydroxy groups confirms the presence of the macrocycle **6** in the *partial cone* conformation.

In the IR spectra of the compounds **4** and **6**, the absorption bands for the stretching vibrations of the hydroxy groups (ν 3330 and 3745 cm^−1^, respectively) were observed. However this absorption band is absent in the IR spectra of compounds **3** and **5**. It confirms complete substitution of the initial thiacalix[4]arenes. It is obvious that the *cone* formation of the thiacalix[4]arenes **3** and **5** is stabilized by intramolecular hydrogen bonding observed in the IR spectra. The bands of stretching vibrations for the NH bonds of *N*-(4’-nitrophenyl)acetamide fragment are observed in the IR spectra of the macrocycles **3** and **5** as a narrow and broadened bands in the region of 3383–3278 cm^−1^ (see Supporting Information File 1, Table S1). In the IR spectra of the compounds **4**, **5** and **6**, absorption bands of valence vibrations for the *N*,*N*-diethylacetamide group (ν 1658, 1648 and 1665 cm^−1^, respectively) were observed, which were absent in the IR spectrum of the tetrasubstituted macrocycle **3** (see Supporting Information File 1, Table S1).

### Complexation properties of the synthesized *p*-*tert*-butylthiacalix[4]arenes towards some single-charged anions

**Figure 1:**
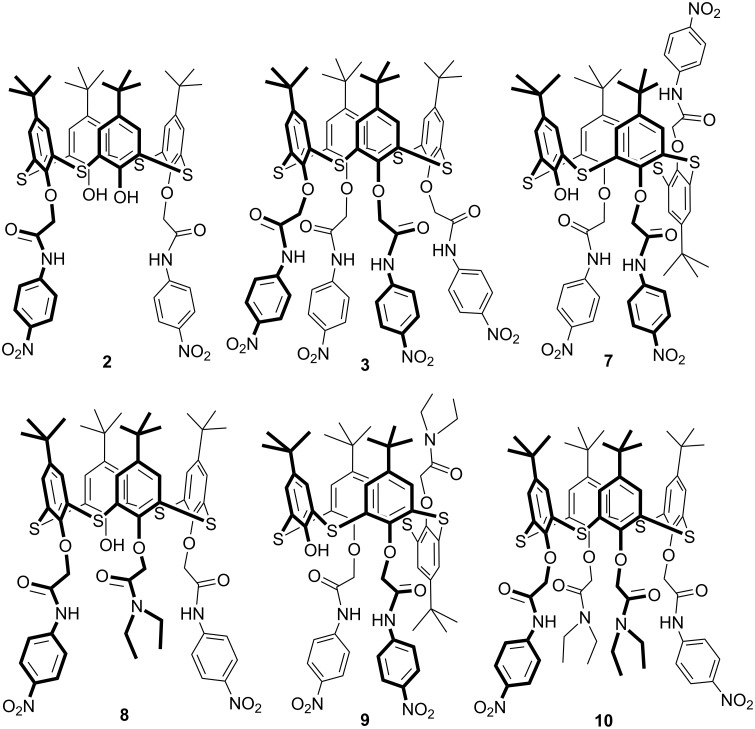
Investigated hosts **2**, **3**, **7**–**10**.

There are several ways of binding negatively charged substrates. Usually, receptors for anions are charged systems capable of electrostatic interaction with anions [1,44–45] or neutral systems using other types of interactions, such as donor–acceptor interactions, hydrogen bonds, hydrophobic effects, etc. [46–57]. Modified macrocycles with the amide fragments at the lower rim can form complexes with the anions by hydrogen bonds of the amide group with the guest. It was shown that (thia)calix[4]arene derivatives with urea and thiourea fragments at the lower rim can bind anions through hydrogen bonds [49,58–61]. In this regard, it was suggested that the synthesized thiacalix[4]arenes **3**, **5** and **6** could be potential receptors for anions because they contain proton donor groups (amide, hydroxy) at the lower rim. Also, the comparison of the complexation properties of early synthesized thiacalix[4]arenes **2**, **7**–**10** (Figure 1) [26,42] with *N*-(4’-nitrophenyl)acetamide and *N*,*N*-diethylacetamide fragments was of interest.

Initially, molecular modeling of the host–guest complexes of the above-mentioned thiacalix[4]arenes **2**, **3**, **5**–**10** with a number of single-charged anions (F^−^, Cl^−^, Br^−^, I^−^, CH_3_CO_2_^−^, H_2_PO_4_^−^, NO_3_^−^) was carried out at a semi-empirical level using the quantum-mechanical method, PM3 (HyperChem 7.0). The proposed model for the binding of anions by *p*-*tert*-butylthiacalix[4]arenes containing proton–donor (amide, hydroxy) groups was studied in order to identify steric and/or electronic hindrances to the complex formation. Review of literature data [62–65] indicate a good agreement between the results of calculations involving the complexes geometry determined by this method and experimental data. The PM3 method is used quite productively for molecular design and modeling of the receptor structures [64–65]. Unfortunately, this calculation method does not provide adequate absolute energy values for the molecules calculated. Therefore, we further discuss the comparative values of the energies of different complexes [66].

As an example, Table 1 shows the difference in the energy gain of the resulting complexes of the macrocycles **3** and **9** with a number of single-charged anions. It can be clearly seen that a linear dependence of the anion size on the energy difference is observed for halide anions. That is, the larger the anion size, the lower the gain in the energy of the complex formed. Thus, the binding efficiency should decrease in the range from the F^−^ ion to the larger I^−^ ion.

**Table 1:** The difference in the energy gain of the complexes of thiacalix[4]arenes **3** and **9** with a number of anions.

compound	Δ*E*, kcal/mol						

	F^−^	Cl^−^	Br^−^	I^−^	H_2_PO_4_^−^	CH_3_CO_2_^−^	NO_3_^−^

**3**	97.32	70.14	58.38	40.93	40.35	42.90	55.69
**9**	93.54	64.77	43.98	14.12	26.66	43.49	18.19

The receptor properties of the synthesized compounds **3**, **5**, **6** toward the tetrabutylammonium salts *n*-Bu_4_NX (X = F^−^, Cl^−^, Br^−^, I^−^, CH_3_CO_2_^−^, H_2_PO_4_^−^, NO_3_^−^) were studied by UV spectroscopy for investigation the influence of structural factors (the macrocycle conformation, the number and the nature of the substituents) on the complexation properties of *p-tert*-butylthiacalix[4]arenes. In these thiacalix[4]arenes, both proton donating secondary amide (*N*-(4’-nitrophenyl)acetamide fragment) and hydroxy (phenolic fragments) groups can participate in anion binding.

To determine the possibility of the tetrabutylammonium cation binding by the synthesized thiacalix[4]arenes, solutions of the compounds **3**, **5** and **6** in the presence of a 10-fold excess of *n*-Bu_4_NX (X = F^−^, Cl^−^, Br^−^, I^−^, CH_3_CO_2_^−^, H_2_PO_4_^−^, NO_3_^−^) were studied in CDCl_3_ by ^1^H NMR spectroscopy. In the ^1^H NMR spectra, the chemical shifts of the *n*-Bu_4_N^+^ protons did not change indicating the absence of interaction between the thiacalix[4]arenes obtained and tetrabutylammonium cation.

The complexation of anions by compounds **3**, **5** and **6** was studied in the presence of a 200-fold excess of *n*-Bu_4_NX in chloroform. The most significant changes in the absorption spectra of *p-tert*-butylthiacalix[4]arenes in the presence of *n*-Bu_4_NX involved the observed bathochromic shift of the absorption band in the 300–370 nm region representing the interaction of the macrocycles studied with *n*-Bu_4_NH_2_PO_4_ and *n*-Bu_4_NF. Besides, changes were observed when the macrocycle **6** interacted with *n*-Bu_4_NCH_3_CO_2_ and in the case of the compound **3**, when *n*-Bu_4_NCl, *n*-Bu_4_NBr, *n*-Bu_4_NI, *n*-Bu_4_NCH_3_CO_2_, and *n*-Bu_4_NNO_3_ were added.

In the absorption spectra of the thiacalix[4]arene **5** with *n*-Bu_4_NX, the changes are observed only in the interaction with F^−^ and H_2_PO_4_^−^ anions. A strong hypochromic effect on the absorption band maximum in both cases and a weak bathochromic shift of the absorption band in the interaction of the macrocycle **5** with F^−^ anions were observed (see Supporting Information File 1, Figure S17).

Interaction of thiacalix[4]arene **6** with the F^−^, CH_3_CO_2_^−^, H_2_PO_4_^−^ anions have a complicated character in the spectra. There is a bathochromic shift and a hypochromic effect on the maximum of the absorption band at 305 nm. In addition, a new absorption maximum appears in the region of 335 nm (see Supporting Information File 1, Figure S18).

**Figure 2:**
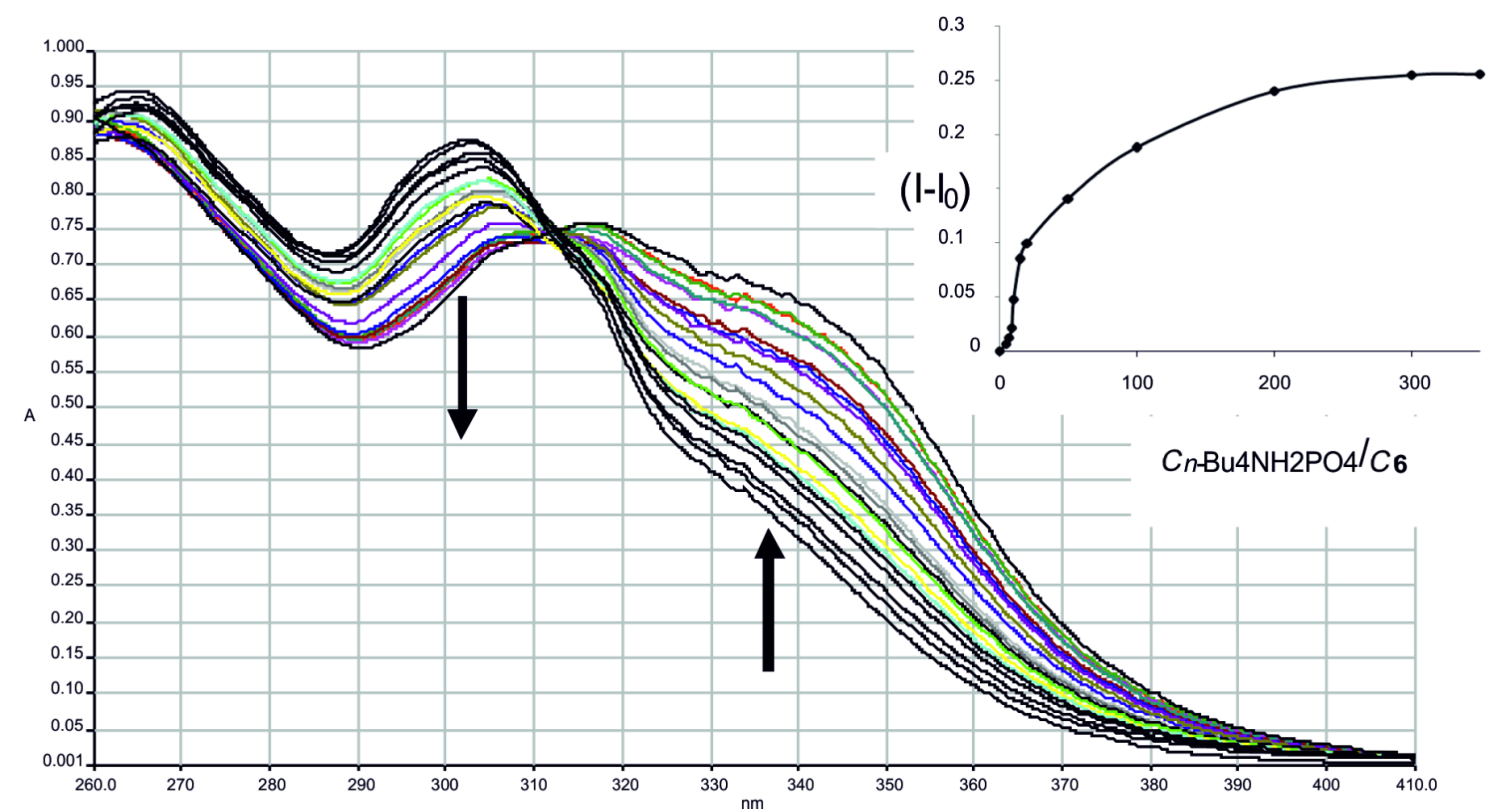
UV absorption spectra of the complexation system obtained by titration of the receptor **6** (*c*(**6**) = 2.5 × 10^−5^ M) and H_2_PO_4_^−^ anion (*c*_initial_ = 2.5 × 10^-5^ M, *c*_final_ = 8.75 × 10^−3^ M) in CHCl_3_. Inset: titration curve (*c*(**6**) = 2.5 × 10^−5^ M).

The complexation ability of the compounds **3**, **5** and **6** in relation to the anions (F^−^, Cl^−^, Br^−^, I^−^, СН_3_СО_2_^−^, Н_2_РО_4_^−^, NO_3_^−^) was quantitatively evaluated by the stoichiometry and the association constants (Table 2) of the formed complexes. They were determined by the isomolar series method, that all the thiacalix[4]arenes studied form in CHСl_3_ the 1:1 complexes with selected *n*-Bu_4_NX (see Supporting Information File 1, Figure S19). The association constants of the complexes studied were estimated by the dilution method (Figure 2). The appropriate complexation constants (Table 2) were determined by the Benesi–Hildebrand method [67].

**Table 2:** The logarithms of the association constant (log*K*_ass_) of the complexes of the synthesized compounds with anions.

compound	[H]:[G]	log*K*_ass_						

		F^−^	Cl^−^	Br^−^	I^−^	H_2_PO_4_^−^	CH_3_CO_2_^−^	NO_3_^−^

**2**	1:1	3.67 ± 0.19^b^	–^b^	–^b^	–^b^	3.41 ± 0.09^b^	3.50 ± 0.32^b^	–^b^
**3**	1:1	5.87 ± 0.23	5.12 ± 0.07	4.51 ± 0.10	3.55 ± 0.13	5.81 ± 0.24	5.90 ± 0.20	3.83 ± 0.12
**5**	1:1	2.53 ± 0.07	–^а^	–^а^	–^а^	2.18 ± 0.38	–^а^	–^а^
**6**	1:1	4.23 ± 0.17	–^а^	–^а^	–^а^	2.90 ± 0.31	3.15 ± 0.30	–^а^
**7**	1:1	5.25 ± 0.56^b^	–^b^	–^b^	–^b^	4.93 ± 0.21^b^	4.85 ± 0.12^b^	–^b^
**8**	1:1	3.53 ± 0.44^b^	–^b^	–^b^	–^b^	–^b^	–^b^	–^b^
**9**	1:1	3.80 ± 0.32^b^	3.36 ± 0.18^b^	2.93 ± 0.04^b^	–^b^	3.51 ± 0.10^b^	3.37 ± 0.11^b^	2.64 ± 0.09^b^

^a^No changes in UV spectra. ^b^The data from ref. [26].

Trisubstituted thiacalix[4]arene **8** in *cone* conformation binds the F^−^ anion with high selectivity against other anions studied. Apparently, the presence of a bulky diethylamide group makes it difficult to bind other anions. However, trisubstituted thiacalix[4]arene **9** in *partial cone* conformation effectively binds almost all the anions studied except the large I^−^ ion.

Among all the macrocycles studied tetrasubstituted at the lower rim *p*-*tert*-butylthiacalix[4]arene **3** containing four *N*-(4’-nitrophenyl)acetamide fragments binds anions most effectively. In some cases, the selectivity of the binding of F^−^, CH_3_CO_2_^−^ and H_2_PO_4_^−^ anions by the macrocycle **3** with respect to I^−^ and NO_3_^−^ anions is significant according to Table 3. Replacement of one *N*-(4’-nitrophenyl)acetamide fragment in the compound **3** by *N*,*N*-diethylacetamide group leads to a significant decrease in the complexation ability of the macrocycle **5**, while the replacement of two fragments leads to the absence of complexation between the thiacalix[4]arene **10** and the anions studied [26]. Compared to the macrocycle **3**, the complexation properties of other thiacalix[4]arenes studied are much worse. However, in some cases (compounds **2**, **5**, **6**, **7**, **8**) a selectivity of binding to F^−^, CH_3_CO_2_^−^ and H_2_PO_4_^−^ anions is observed in the series of the single-charged anions studied.

**Table 3:** The selectivity of binding to F^−^, Cl^−^, H_2_PO_4_^−^ and CH_3_CO_2_^−^ anions by macrocycle **3** in comparison with Br^−^, I^−^ and NO_3_^−^ anions.

	Selectivity = *K*_guest_**_1_**/*K*_guest_**_2_** (**1**)			

guest **1**	F^−^	Cl^−^	H_2_PO_4_^−^	CH_3_CO_2_^−^

guest **2**				
Br^−^	22.91	4.07	19.95	24.55
I^−^	208.93	37.15	181.97	223.87
NO_3_^−^	109.65	19.50	95.50	117.50

It is interesting to note that 1,2-disubstituted *p*-*tert*-butylthiacalix[4]arene **6** in *partial cone* conformation containing only one amide fragment binds to F^−^, CH_3_CO_2_^−^ and H_2_PO_4_^−^ ions. Probably, the aromatic rings of the macrocycle with free phenolic groups are arranged in such a manner that the hydroxy groups are located near the amide thereby forming a cavity complementary to the anions studied. In this case, macrocycle **6** selectively binds to F^−^ anions, in contrast to the 1,3-disubstituted macrocycle **2** containing two proton donor fragments. The selectivity of binding to F^−^ anions compared to H_2_PO_4_^−^ and CH_3_CO_2_^−^ anions is 21.4 and 12.0, respectively.

The comparison of the experimental data obtained (logarithms of the association constants of the complexes of the studied thiacalix[4]arenes with a series of anions) and the results of quantum mechanical calculations (energy change in the formation of guest–host complexes calculated by the PM3 method) (HyperChem 7.0) was the final stage of the work (Table 4).

**Table 4:** The logarithms of the association constants (log*K*_ass_) of the complexes of the thiacalix[4]arenes **2**, **3**, **5**–**9** with anions and the energy change in the formation of guest–host complexes calculated by the PM3 method.

Compound		**2**	**3**	**5**	**6**	**7**	**8**	**9**

F^−^	log*K*_ass_	3.67	5.87	2.53	4.23	5.25	3.53	3.80
	Δ*E*	86.8	97.3	108.1	81.6	114.0	93.2	93.5
Cl^−^	log*K*_ass_	–	5.12	–	–	–	–	3.36
	Δ*E*	–	70.1	–	–	–	–	64.8
Br^−^	log*K*_ass_	–	4.51	–	–	–	–	2.93
	Δ*E*	–	58.4	–	–	–	–	44.0
I^−^	log*K*_ass_	–	3.55	–	–	–	–	–
	Δ*E*	–	40.9	–	–	–	–	14.1
H_2_PO_4_^−^	log*K*_ass_	3.41	5.81	2.18	2.90	4.93	–	3.51
	Δ*E*	40.2	40.4	29.6	31.9	23.9	31.44	26.7
CH_3_CO_2_^−^	log*K*_ass_	3.50	5.90	–	3.15	4.85	–	3.37
	Δ*E*	44.5	42.9	35.8	40.1	27.8	30.0	43.5
NO_3_^−^	log*K*_ass_	–	3.83	–	–	–	–	2.64
	Δ*E*	–	55.7	–	–	–	–	18.2

The results of the semi-empirical modeling are in a good agreement with spectrophotometric titration data in the case of halide ions: the efficiency of the anion binding by thiacalix[4]arenes **3** and **9** decreases in the range from F^−^ ion to the larger I^−^ ion (Table 4). In the case of the macrocycles **3** and **9**, the logarithm of the association constant of the resulting complexes increases from the energy gain difference calculated using the semi-empirical PM3 method (Table 4). However, an unambiguous tendency is observed only for spherical substrates (halide ions). But there is no such dependence in the case of tetrahedral H_2_PO_4_^−^, Y-shaped CH_3_CO_2_^−^ and NO_3_^−^ ions.

It is clearly seen from the Table 4 that all the studied thiacalix[4]arenes (**2**, **3**, **5**–**9**) bind F^–^ ions. This agrees well with the quantum mechanical calculations. For all the complexes involving the macrocycles studied with F^−^ ion, a significant energy gain within the range of 82–114 kcal/mol is observed.

The quantum mechanical calculations of the complexes of the thiacalix[4]arenes **2**, **3**, **6**, **7**, **9** with CH_3_CO_2_^−^ ion are in good agreement with the logarithms of the association constant (Table 4). An increase in the logarithm of the association constant correlates with an increase in the energy change in the formation of the host–guest complexes in the case of the macrocycles **3**, **7**. For the compounds **2**, **6**, **9**, the efficiency of complexation is close.

## Conclusion

New mono-, 1,2-di- and tetrasubstituted at the lower rim *p*-*tert*-butylthiacalix[4]arenes containing *N*-(4’-nitrophenyl)acetamide and *N*,*N*-diethylacetamide groups in *cone* and *partial cone* conformations were synthesized. The calculations of the proposed model for the anion binding by the synthesized thiacalix[4]arenes were carried out using molecular modeling (the quantum mechanical method PM3). Also, their complexation ability toward a number of tetrabutylammonium salts *n*-Bu_4_NX (X = F^−^, Cl^−^, Br^−^, I^−^, CH_3_CO_2_^−^, H_2_PO_4_^−^, NO_3_^−^) was studied by UV spectroscopy. Using *p-tert*-butylthiacalix[4]arenes substituted at the lower rim by *N*-(4’-nitrophenyl)acetamide and *N*,*N*-diethylacetamide as an example, the influence of the number and nature of substituents on the complexation properties of the synthetic receptors based on thiacalix[4]arene was demonstrated. The most effective receptor, i.e., *p*-*tert*-butylthiacalix[4]arene tetrasubstituted at the lower rim by *N*-(4’-nitrophenyl)acetamide in *cone* conformation binds to the anions studied with the association constants in the range of 3.55 × 10^3^–7.94 × 10^5^ M^−1^, and the selectivity of binding to F^−^, Cl^−^, CH_3_CO_2_^−^, H_2_PO_4_^−^ anions aginst other anions is in the range of 4.1–223.9. The 1,2-disubstituted thiacalix[4]arene in the *partial cone* conformation containing only one *N*-(4’-nitrophenyl)acetamide fragment selectively binds to F^−^ anion in contrast to the 1,3-disubstituted macrocycle containing two such fragments. These novel anion receptors based on the synthesized compounds can be used in the development of systems like an "electronic tongue", biomimetic materials and diagnostic agents.

## Supporting Information

File 1 Additional experimental parameters and results.
