# Supplementary material for: p-tert-Butylthiacalix[4]arenes functionalized by N-(4’-nitrophenyl)acetamide and N,N-diethylacetamide fragments: synthesis and binding of anionic guests
Source: Beilstein J Org Chem. 2017 Sep 13;13:1940–9. doi: 10.3762/bjoc.13.188 (PMC5629390; doi:10.3762/bjoc.13.188)
Supplement: File 1 — Additional experimental parameters and results. [file Beilstein_J_Org_Chem-13-1940-s001.pdf]

Supporting Information

for

***p-tert*-Butylthiacalix[4]arenes functionalized by *N*-(4'-nitrophenyl)acetamide and *N*,*N*-diethylacetamide fragments: synthesis and binding of anionic guests**

Alena A. Vavilova and Ivan I. Stoikov*

Address: Kazan Federal University, 420008 Kremlevskaya, 18, Kazan, Russian Federation

Email: Ivan Ivanovich Stoikov - Ivan.Stoikov@mail.ru

*Corresponding author

Additional experimental parameters and results

Table S1: The IR spectra data of thiacalix[4]arenes **3–6** (ν/cm^{-1}).

**Groups**	**3**	**4**	**5**	**6**
**-OH**	-	3330	-	3745
**-NH-**	3345, 3278	-	3383, 3285	3380, 3299
**-C(O)-NEt$_2$**	-	1658	1648	1665
**-C(O)-NH-**	1707, 1615, 1597	-	1697, 1543, 1307	1672, 1541, 1301

Experimental

General

Melting points were determined using a Boetius Block apparatus. All chemicals were purchased from Aldrich and most of them were used as received without additional purification. Organic solvents were purified by standard procedures. ^{1}H NMR spectra were recorded at room temperature with a 300 MHz Varian XL-300 spectrometer in $CDCl_3$ or $CDCl_3$/DMSO-d_6 as the solvent; chemical shifts are reported in ppm. The 2D and ^{13}C NMR spectra were recorded on a Bruker-500 MHz instrument in $CDCl_3$ at room temperature. IR spectra (nujol) were recorded with a Vector 22 (Bruker) IR spectrometer. Mass spectra were recorded with a Bruker Esquire MS, MALDI–TOF Dynamo Finnigan (with 1,8,9-trihydroxyanthracene or 4-nitroaniline matrices), and a Varian MAT 312. Elemental analyses were performed with Perkin–Elmer 2400 Series II instruments.

The investigations of the receptor abilities of the thiacalix[4]arene derivatives **3**, **5**, **6** were carried out in $CHCl_3$ (analytical grade). UV spectra were registered on a Perkin Elmer Lambda 35 spectrophotometer.

5,11,17,23-Tetra-*tert*-butyl-25,27-dihydroxy-26,28-bis[*N*-(4’-nitrophenyl)-aminocarbonylmethoxy]-2,8,14,20-tetrathiacalix[4]arene (*cone* 2) was obtained according to the literature procedure [1], Mp: 315°C.

5,11,17,23-Tetra-*tert*-butyl-25-hydroxy-26,27,28-tris[*N*-(4’-nitrophenyl)-aminocarbonylmethoxy]-2,8,14,20-tetrathiacalix[4]arene (*partial cone* 7) was obtained according to the literature procedure [1], Mp: 211°C.

5,11,17,23-Tetra-*tert*-butyl-25-hydroxy-26,28-bis[*N*-(4’-nitrophenyl)-aminocarbonylmethoxy]-27-[*N,N*-diethylcarbamoylmethoxy]-2,8,14,20-

tetrathiacalix[4]arene (*cone* 8) was obtained according literature procedure [2], Mp: 256 °C.

5,11,17,23-Tetra-*tert*-butyl-25-hydroxy-26,28-bis[*N*-(4'-nitrophenyl)-aminocarbonylmethoxy]-27-[*N,N*-diethylcarbamoylmethoxy]-2,8,14,20-tetrathiacalix[4]arene (*partial cone* 9) was obtained according to the literature procedure [2], Mp: 168 °C.

5,11,17,23-Tetra-*tert*-butyl-25,27-bis[*N*-(4'-nitrophenyl)-aminocarbonylmethoxy]-26,28-bis[*N,N*-diethylcarbamoylmethoxy]-2,8,14,20-tetrathiacalix[4]arene (*cone* 10) was obtained according to the literature procedure [2], Mp: 183 °C.

5,11,17,23-Tetra-*tert*-butyl-25,26,27,28-tetrakis[*N*-(4'-nitrophenyl)-aminocarbonylmethoxy]-2,8,14,20-tetrathiacalix[4]arene (*cone* 3). A mixture of 5.00 g (6.94 mmol) of 5,11,17,23-tetra-*tert*-butyl-25,26,27,28-tetrahydroxy-2,8,14,20-tetrathiacalix[4]arene (**1**), 7.19 g (27.77 mmol) of 2-bromo-*N*-(4'-nitrophenyl)acetamide and 2.94 g (27.77 mmol) of anhydrous sodium carbonate in 180 ml of dry acetonitrile was refluxed for 40 hours. After cooling the precipitate was filtered from the reaction mixture, dissolved in chloroform and washed with 2 M HCl. The organic layer was separated and dried over molecular sieves 3 Å. The solvent was removed on a rotary evaporator. The recrystallization of the resulting solid from chloroform/ethyl alcohol mixture gave a pure sample of 1,3-disubstituted derivative **2** (colorless crystals) in 4.48 g (60%) yield. The filtrate was evaporated under reduced pressure. The residue formed was filtered and dried in vacuo over P_2O_5. Thiacalix[4]arene **3** in *cone* conformation was obtained in yield of 1.98 g (10%). Mp: 185°C. ^{1}H NMR ($CDCl_3$/DMSO-d_6, δ, ppm, J/Hz): 1.06 (s, 36H, $(CH_3)_3C$); 5.02 (s, 8H, -O-CH_2-); 7.34 (s, 8H, Ar-H); 7.75 (AB part of AA'BB' system, 8H, Ar'-H, $^3J_{AB}+^5J_{AB'}$ =9.2

Hz); 7.93 (A'B' part of AA'BB' system, 8H, Ar'-H, $^{3}J_{AB}$ +$^{5}J_{AB'}$ =9.2 Hz); 10.50 (s, 4H, NH). ^{13}C NMR ($CDCl_3$/DMSO-d_6, δ, ppm): 30.71, 33.82, 73.63, 119.23, 124.16, 127.61, 134.37, 142.51, 143.85, 146.77, 157.38, 167.56. ^{1}H-^{1}H NOESY NMR spectrum (most important cross-peaks are presented): H^{4b}/H^{5}, H^{7}/H^{9}, H^{9}/H^{11}, H^{11}/H^{12}. IR (Nujol, ν/cm^{-1}): 3345, 3278 (NH); 1707 (C=O); 1615, 1597 (C(O)-NH); 1542, 1510, 1377, 1344 (NO_2). MS (MALDI-TOF): calculated [M^{+}] m/z = 1432.4, found $[M+Na]^{+}$ m/z = 1455.5. El. Anal. Calcd for $C_{72}H_{72}N_8O_{16}S_4$ (%): C, 60.32; H, 5.06; N, 7.82; S, 8.95. Found (%):C, 59.93; H, 4.87; N, 7.81; S, 8.24.

5,11,17,23-Tetra-*tert*-butyl-25,26,27-trihydroxy-28-[*N,N*-diethylcarbamoylmethoxy]-2,8,14,20-tetrathiacalix[4]arene (*cone* 4).

Procedure A. The mixture of 10.00 g (13.89 mmol) of 5,11,17,23-tetra-*tert*-butyl-25,26,27,28-tetrahydroxy-2,8,14,20-tetrathiacalix[4]arene (**1**), 18.11 g (55.56 mmol) of anhydrous cesium carbonate and 3.90 ml (27.78 mmol) of 2-chloro-*N*,*N*-diethylacetamide in 300 ml of dry acetone was refluxed for 60 hours. After cooling, the precipitate from the reaction mixture was filtered. The filtrate was concentrated under reduced pressure. The residue was dissolved in chloroform and washed with 2 M HCl. The organic layer was separated, dried over molecular sieves 3 Å. The solvent was removed on a rotary evaporator and methyl alcohol was added. The residue formed was filtered. The monosubstituted derivative **4** (white powder) was obtained with yield of 2.16 g (20%). Mp: 215 ° C.

Procedure B. The mixture of 5.00 g (6.90 mmol) of 5,11,17,23-tetra-*tert*-butyl-25,26,27,28-tetrahydroxy-2,8,14,20-tetrathiacalix[4]arene (**1**), 24.79 g (179.40 mmol) of anhydrous potassium carbonate and 8.44 ml (55.20 mmol) of 2-chloro-*N*,*N*-diethylacetamide in 200 ml of dry acetone was refluxed for 30 hours. After cooling, the precipitate from the reaction mixture was filtered. The filtrate was concentrated under reduced pressure. The residue was dissolved in chloroform

and washed with 2 M HCl. The organic layer was separated, dried over molecular sieves 3 Å. The solvent was removed on a rotary evaporator and methyl alcohol was added. The residue formed was filtered. The monosubstituted derivative **4** (white powder) was obtained with 2.20 g (38%) yield. Mp: 215 ° C. ^{1}H NMR ($CDCl_3$, δ, ppm, *J*/Hz): 1.08 (s, 9H, $(CH_3)_3C$), 1.21 (s, 27H, $(CH_3)_3C$), 1.26 (t, 3H, -CH_3, $^{3}J_{HH}$=7.0 Hz), 1.30 (t, 3H, -CH_3, $^{3}J_{HH}$=7.0 Hz), 3.42 (m, 2H, -$\underline{CH_2}CH_3$), 3.58 (m, 2H, -$\underline{CH_2}CH_3$), 5.31 (s, 2H,-O-CH_2-), 7.46 (s, 2H, Ar-H), 7.56, 7.62 (AB system, 4H, Ar-H_A, Ar-H_B, $^{4}J_{HH}$=2.6 Hz), 7.59 (s, 2H, Ar-H), 9.72 (s, 1H, OH), 9.83 (s, 2H, OH). ^{13}C NMR ($CDCl_3$, δ, ppm): 13.05; 14.29; 30.96; 31.29; 34.06; 40.36; 41.18; 72.55; 76.75; 77.25; 120.82; 120.87; 121.32; 128.84; 135.83; 135.94; 136.30; 143.27; 156.78; 168.11. ^{1}H-^{1}H NOESY NMR spectrum (most important cross-peaks are presented): H^3/H^{4b}, $H^{3'}/H^{4'b}$, $H^{3'}/H^5$, $H^7/H^{7'}$, H^7/H^{7+}. IR (Nujol, ν/cm^{-1}): 3330 (OH); 1658 (C(O)-NEt_2). MS (MALDI-TOF): calculated [M^+] m/z = 833.3, found $[M+H]^+$ m/z = 834.7, $[M+Na]^+$ m/z = 856.8, $[M+K]^+$ m/z = 872.6. El. Anal. Calcd for $C_{46}H_{59}NO_5S_4$ (%): C, 66.23; H, 7.13; N, 1.68. Found (%): C, 65.93; H, 7.46; N, 1.72.

5,11,17,23-Tetra-*tert*-butyl-25,26,27-tris[*N*-(4’-nitrophenyl)-aminocarbonylmethoxy]-28-[*N,N*-diethylcarbamoylmethoxy]-2,8,14,20-tetrathiacalix[4]arene (*cone* 5). A mixture of 2.00 g (2.40 mmol) of 5,11,17,23-tetra-*tert*-butyl-25,26,27-trihydroxy-28-[*N*,*N*-diethylcarbamoylmethoxy]-2,8,14,20-tetrathiacalix[4]arene (**4**), 1.52 g (14.40 mmol) of anhydrous sodium carbonate and 3.72 g (14.40 mmol) of 2-bromo-*N*-(4’-nitrophenyl)acetamide in 70 ml of dry acetone was refluxed for 50 hours. After cooling, the precipitate from the reaction mixture was filtered, dissolved in chloroform and washed with 2M HCl. The organic layer was separated, dried over molecular sieves 3 Å. The solvent was removed on a rotary evaporator. 40 ml of ethyl alcohol was added to the residue.

The resulting pale yellow precipitate was filtered and recrystallized from chloroform/ethyl alcohol mixture. Thiacalix[4]arene **5** was obtained in 1.41 g (43%) yield. Mp: 215 ° C. ^{1}H NMR ($CDCl_3$, δ, ppm, *J*/Hz): 0.97 (s, 9H, $(CH_3)_3C$), 0.98 (s, 9H, $(CH_3)_3C$), 1.11 (t, 6H, $-CH_3$; $^{3}J_{HH}$=7.1 Hz), 1.31 (s, 18H, $(CH_3)_3C$), 3.06 (m, 2H, -N$\underline{CH_2}$$CH_3$), 3.23 (m, 2H, -N$\underline{CH_2}$$CH_3$), 4.63 (s, 2H, -O-$CH_2$-), 4.88 (s, 2H, -O-$CH_2$-), 5.40 (d of AB system, 2H, -O-CH_2-C(O)NH, $^{2}J_{HH}$=15.9 Hz), 5.58 (d of AB system, 2H, -O-CH_2-C(O)NH, $^{2}J_{HH}$=15.9 Hz), 7.07 (s, 2H, Ar-H), 7.14 (s, 2H, Ar-H), 7.66 (d of AB system, 2H, Ar-H_A, $^{4}J_{HH}$=2.6 Hz), 7.70 (d of AB system, 2H, Ar-H_B, $^{4}J_{HH}$=2.6 Hz), 7.78 (AB part of AA'BB' system, 4H, Ar'-H, $^{3}J_{AB}$ +$^{5}J_{AB'}$ =9.1 Hz), 8.01 (AB part of AA'BB' system, 2H, Ar'-H, $^{3}J_{AB}$ +$^{5}J_{AB'}$ =8.8 Hz), 8.04 (A'B' part of AA'BB' system, 4H, Ar'-H, $^{3}J_{AB}$ +$^{5}J_{AB'}$ =9.1 Hz), 8.16 (A'B' part of AA'BB' system, 2H, Ar'-H, $^{3}J_{AB}$ +$^{5}J_{AB'}$ =8.8 Hz), 10.56 (s, 2H, NH); 10.58 (s, 1H, NH). ^{13}C NMR ($CDCl_3$, δ, ppm): 12.64; 14.09; 30.88; 31.27; 34.41; 73.47; 76.75; 77.25; 119.41; 120.15; 124.63; 126.95; 127.96; 133.43; 136.12; 143.38; 144.02; 147.28; 158.33; 168.06. ^{1}H-^{1}H NOESY NMR spectrum (most important cross-peaks are presented): H^3/H^{4b}, $H^{3'}/H^{4'b}$, H^{3+}/H^{4+b}, $H^7/H^{7'}$, $H^{7'}/H^{7+}$, $H^5/H^{3'}$, $H^{5'}/H^{3+}$, $H^9/H^{9'}$, H^9/H^{10}, $H^{11'}/H^{12'}$. IR (Nujol, ν/cm^{-1}): 3383, 3285 (NH); 1697, 1543, 1307 (C(O)-NH); 1648 (-NEt_2). MS (MALDI-TOF): calculated [M^+] m/z = 1367.4, found $[M+Na]^+$ m/z = 1390.6. El. Anal. Calcd for $C_{70}H_{77}N_7O_{14}S_4$ (%): C, 61.43; H, 5.67; N, 7.16; S 9.37. Found (%): C, 61.21; H, 5.19; N, 6.78; S, 9.97.

5,11,17,23-Tetra-*tert*-butyl-25,26-dihydroxy-27-[*N,N*-diethylcarbamoylmethoxy]-28-[*N*-(4'-nitrophenyl)-aminocarbonylmethoxy]-2,8,14,20-tetrathiacalix[4]arene (*partial cone* 6). The mixture of 0.50 g (0.60 mmol) of 5,11,17,23-tetra-*tert*-butyl-25,26,27-trihydroxy-28-[*N*,*N*-diethylcarbamoylmethoxy]-2,8,14,20-tetrathiacalix[4]arene (**4**), 1.17 g (3.60 mmol) of anhydrous cesium carbonate and 0.93 g (3.60 mmol) of 2-bromo-*N*-(4'-

nitrophenyl)acetamide in 50 ml of dry acetone was refluxed for 50 hours. After cooling, the precipitate was filtered from the reaction mixture, the filtrate was removed on a rotary evaporator, the residue was dissolved in chloroform and washed with 2 M HCl. The organic layer was separated and dried over molecular sieves 3 Å. The solvent was removed on a rotary evaporator and ethyl alcohol was added. The resulting white precipitate was filtered. The recrystallization of this solid from chloroform/ethyl alcohol mixture gave pure sample of thiacalix[4]arene **6** in 0.35 g (42%) yield. Mp: 195 °C. ^{1}H NMR ($CDCl_3$, δ, ppm, J/Hz): 0.70 (s, 9H, $(CH_3)_3C$), 0.71 (s, 9H, $(CH_3)_3C$), 1.11 (t, 3H, $-CH_3$, $^3J_{HH}$=7.0 Hz), 1.22 (t, 3H, $-CH_3$, $^3J_{HH}$=7.0 Hz), 1.36 (s, 9H, $(CH_3)_3C$), 1.45 (s, 9H, $(CH_3)_3C$), 3.21 (m, 2H, -N<u>CH$_2$</u>CH$_3$), 3.41 (m, 2H, -N<u>CH$_2$</u>CH$_3$), 4.26 (d of AX system, 1H, -O-<u>CH$_2$</u>-NEt$_2$, $^2J_{HH}$=13.8 Hz), 4.58 (d of AB system, 1H, -O-<u>CH$_2$</u>-(CO)NH, $^2J_{HH}$=14.7 Hz), 4.69 (d of AB system, 1H, -O-<u>CH$_2$</u>-C(O)NH, $^2J_{HH}$=14.7 Hz), 5.20 (d of AX system, 1H, -O-<u>CH$_2$</u>-NEt$_2$, $^2J_{HH}$=13.8 Hz), 6.29 (AB part of AA'BB' system, 2H, Ar'-H, $^3J_{AB}+^5J_{AB'}$ =9.4 Hz), 6.31 (s, 1H, NH), 7.07 (d of AB system, 1H, Ar-H, $^4J_{HH}$=2.4 Hz), 7.17 (d of AB system, 1H, Ar-H, $^4J_{HH}$=2.4 Hz), 7.29 (d of AB system, 1H, Ar-H, $^4J_{HH}$=2.4 Hz), 7.34 (d of AB system, 1H, Ar-H, $^4J_{HH}$=2.4 Hz), 7.40 (d of AB system, 1H, Ar-H, $^4J_{HH}$=2.4 Hz), 7.60 (d of AB system, 1H, Ar-H, $^4J_{HH}$=2.4 Hz), 7.66 (A'B' part of AA'BB' system, 2H, Ar'-H, $^3J_{AB}+^5J_{AB'}$ =9.4 Hz), 7.73 (d of AB system, 1H, Ar-H, $^4J_{HH}$=2.4 Hz), 7.82 (d of AB system, 1H, Ar-H, $^4J_{HH}$=2.4 Hz), 8.57 (s, 1H, OH), 10.06 (s, 1H, OH). ^{13}C NMR ($CDCl_3$, δ, ppm): 13.01; 14.43; 30.54; 30.76; 31.30; 31.48; 33.64; 34.65; 40.14; 40.50; 64.57; 70.21; 118.46; 122.20; 124.25; 128.62; 129.32; 129.56; 133.82; 134.37; 134.50; 142.28; 142.71; 143.97; 148.99; 155.68; 156.20; 156.74; 165.80; 166.02. ^{1}H-^{1}H NOESY NMR spectrum (most important cross-peaks are presented): H^3/H^{4b}, $H^{3'}/H^{4'b}$, H^{3+}/H^{4+b}, H^{3*}/H^{4*b}, $H^{9'}/H^{4b}$, $H^{9'}/H^{4*b}$, $H^{11'}/H^{4b}$, $H^{12'}/H^{4b}$, $H^{14'}/H^{4*b}$, $H^{15'}/H^{4*b}$,

$H^{7'}/H^{4b}$, $H^{7'}/H^{4*b}$, H^{7+}/H^{7*}, $H^{7}/H^{3'}$, $H^{7}/H^{4'b}$, H^{7}/H^{9}, H^{9}/H^{10}. IR (Nujol, v/cm^{-1}): 3745 (OH); 3380, 3299 (NH); 1672, 1541, 1301 (C(O)-NH); 1665 ($-NEt_2$). MS (MALDI-TOF): calculated [M^+] m/z = 1011.4, found $[M+Na]^+$ m/z = 1034.5, $[M+K]^+$ m/z = 1050.5. El. Anal. Calcd for $C_{54}H_{65}N_3O_8S_4$ (%): C, 64.07; H, 6.47; N, 4.15; S 12.64. Found (%): C, 64.19; H, 6.41; N, 4.20; S 12.44.

Determination of the stoichiometry by the isomolar series method. The stoichiometry was defined by plotting the curves of isomolar series. For this purposes an array of experiments with constant total concentration of the guest and host molecules (0.02 M) was carried out. A guest/host ratio was varied. Obtained data were processed as described earlier [3]

Experimental methods of determination of the association constant.

Absorption properties of obtained compounds were studied in the chloroform solution (2.5×10^{-5} M). The efficiency of anion binding was estimated by addition of 200-fold excess of tetrabutylammonium salt in chloroform. Concentration of the anion in titration experiment was varied from 2.50×10^{-5} M to 8.75×10^{-3} M. The values of the association constants were determined from the intensity change of the absorption wavelength maximum having the highest hyperchromic effect upon complexation, for which absorption intensity change was the maximum. Also the calculation of the association constants was carried out according the change of the ratio of two wavelengths intensities having a maximum hypo- and hyperchromic effects in complexation [3].

The association constants of the complexes were calculated by the equation $\beta = \alpha \times C_h/[(C_h - a \times \alpha \times C_h)^a \times (C_g - b \times \alpha \times C_h)^b]$, where C_g is the anion concentration, C_h is the receptor concentration, α is the saturation constant of the complex, a is the stoichiometric coefficient of the receptor molecule, b is the stoichiometric coefficient of the salt molecule.

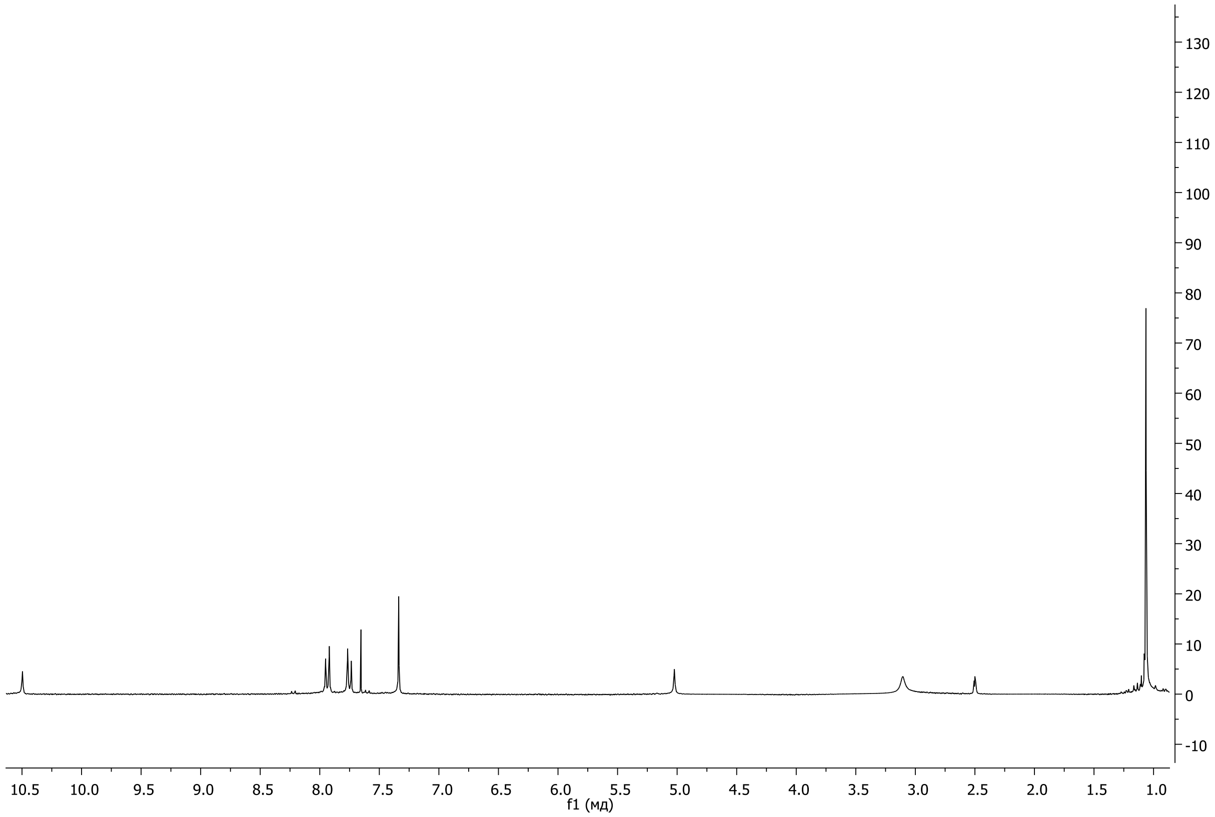

Figure S1: ^{1}H NMR spectrum of compound **3** ($CDCl_3$/DMSO-d_6, 298 K, 300 MHz)

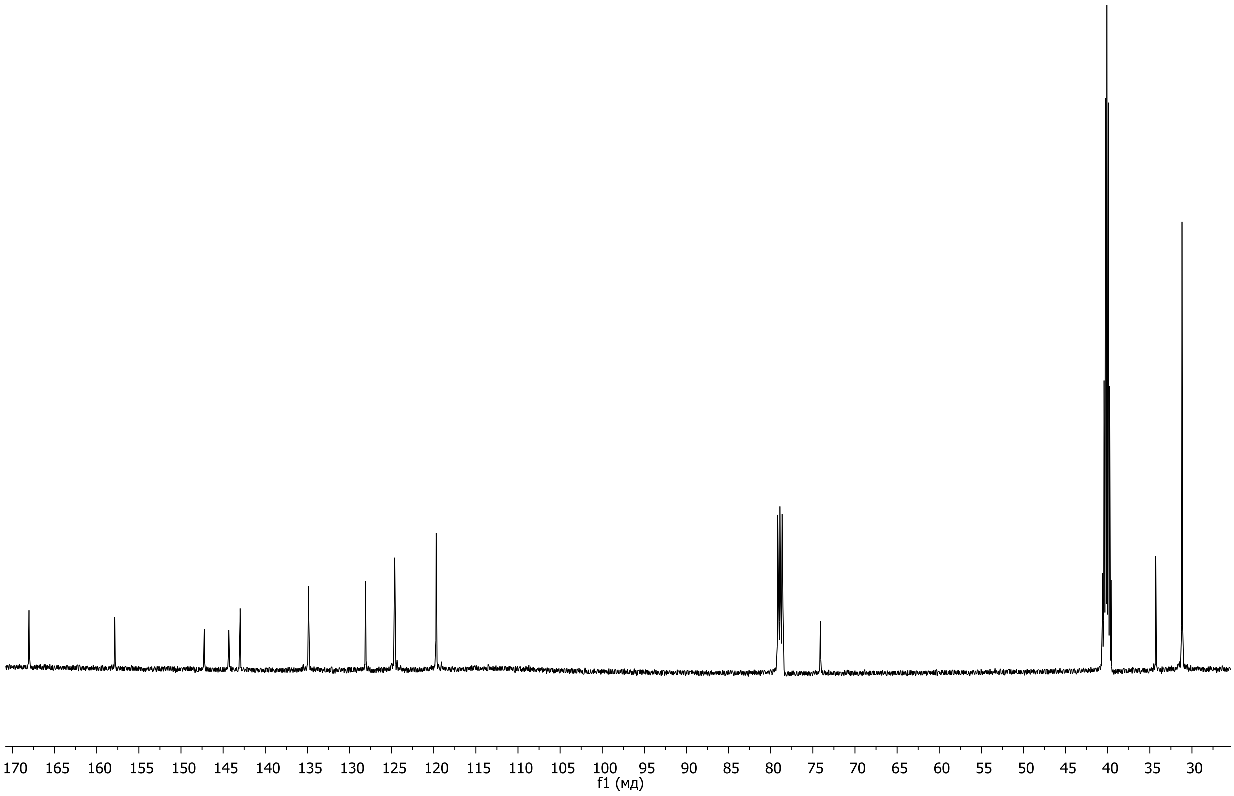

Figure S2: ^{13}C NMR spectrum of compound **3** ($CDCl_3$/DMSO-d_6, 298 K, 125 MHz).

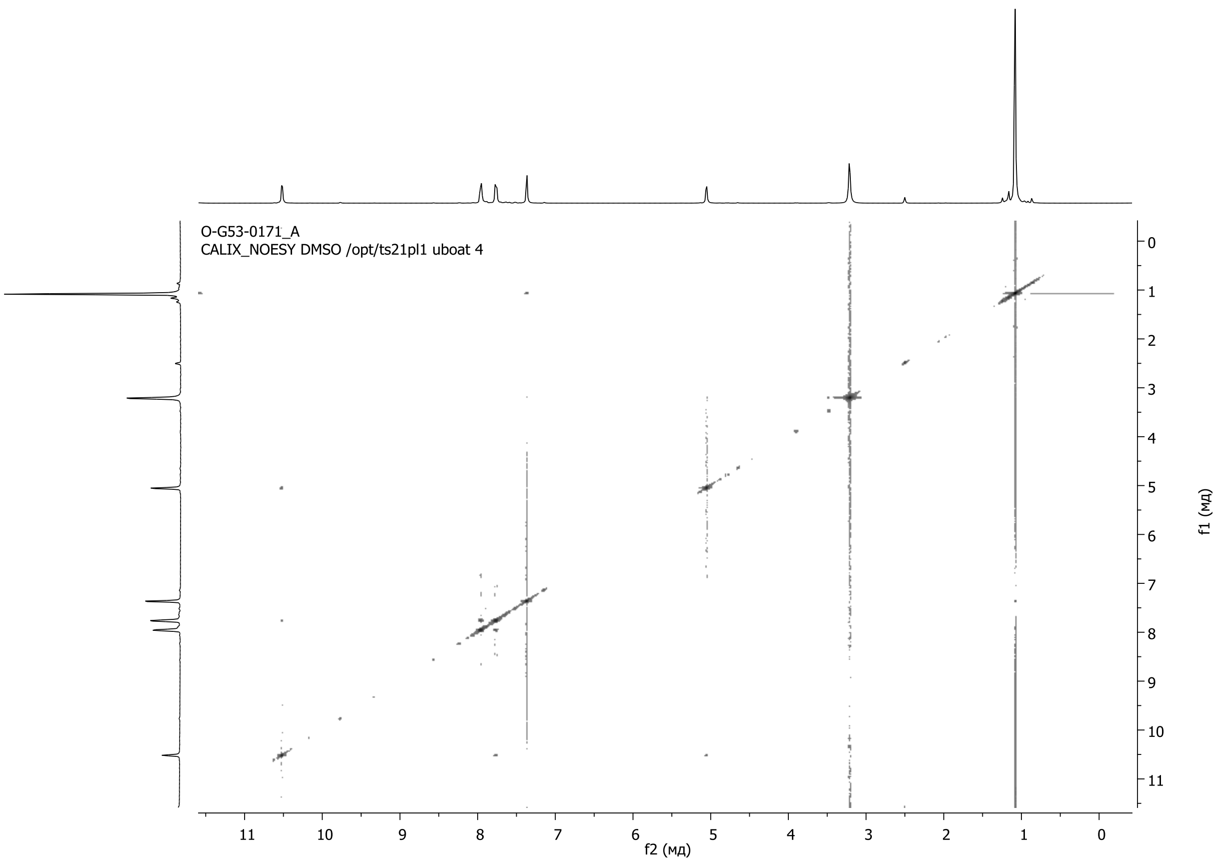

Figure S3: The 2D NMR ^1H-^1H NOESY spectrum of compound **3** (DMSO-d_6, 298 K, 500 MHz).

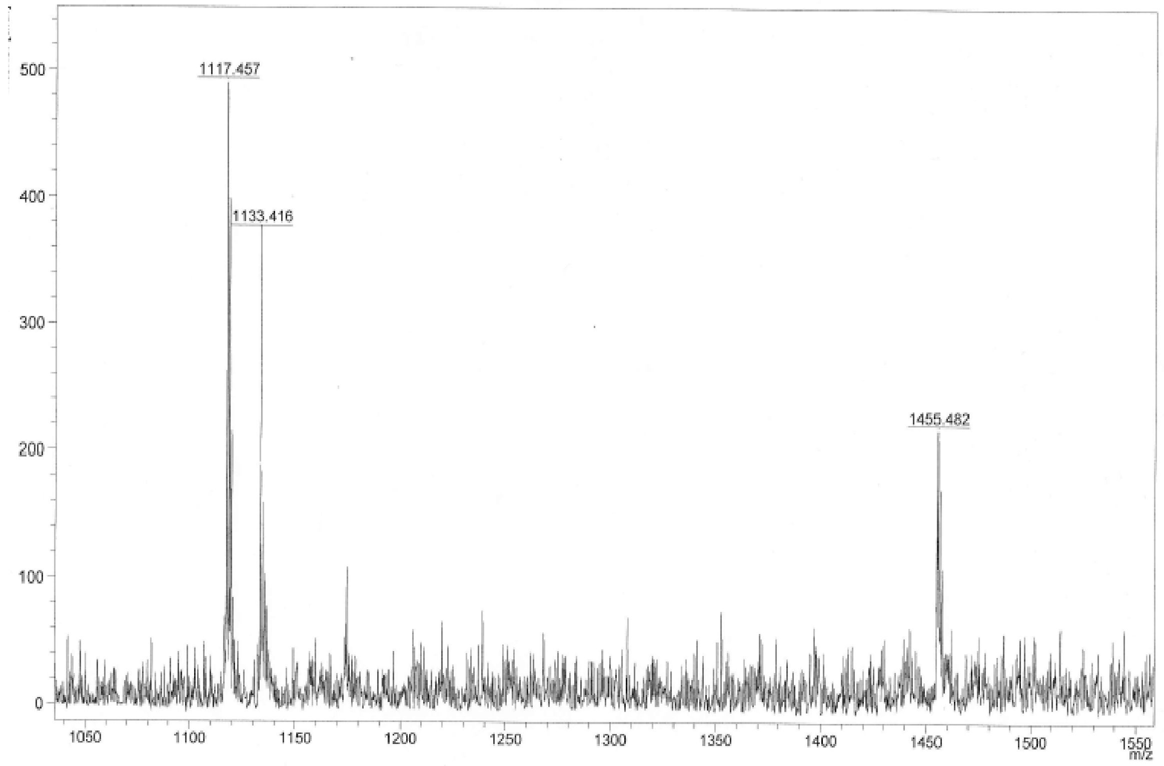

Figure S4: MALDI–TOF mass spectrum of compound **3**.

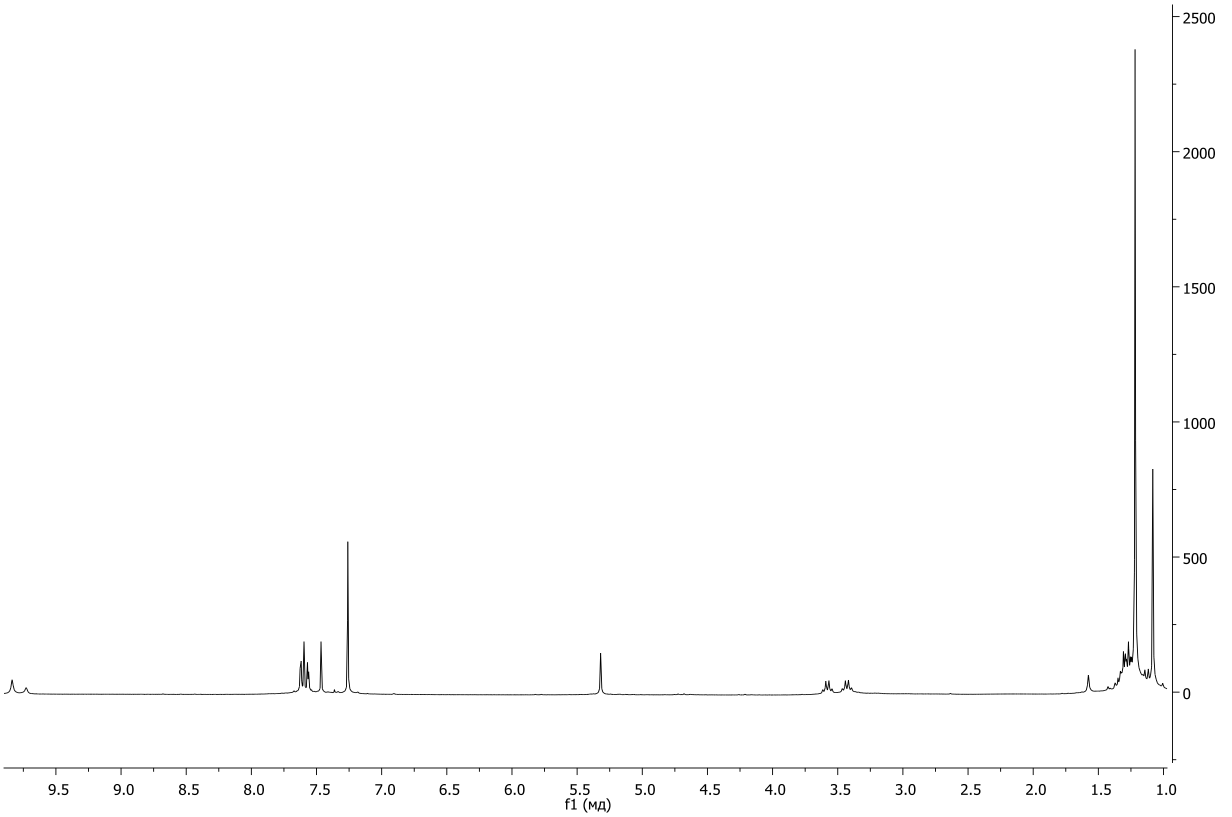

Figure S5: ^{1}H NMR spectrum of compound **4** ($CDCl_3$, 298 K, 300 MHz).

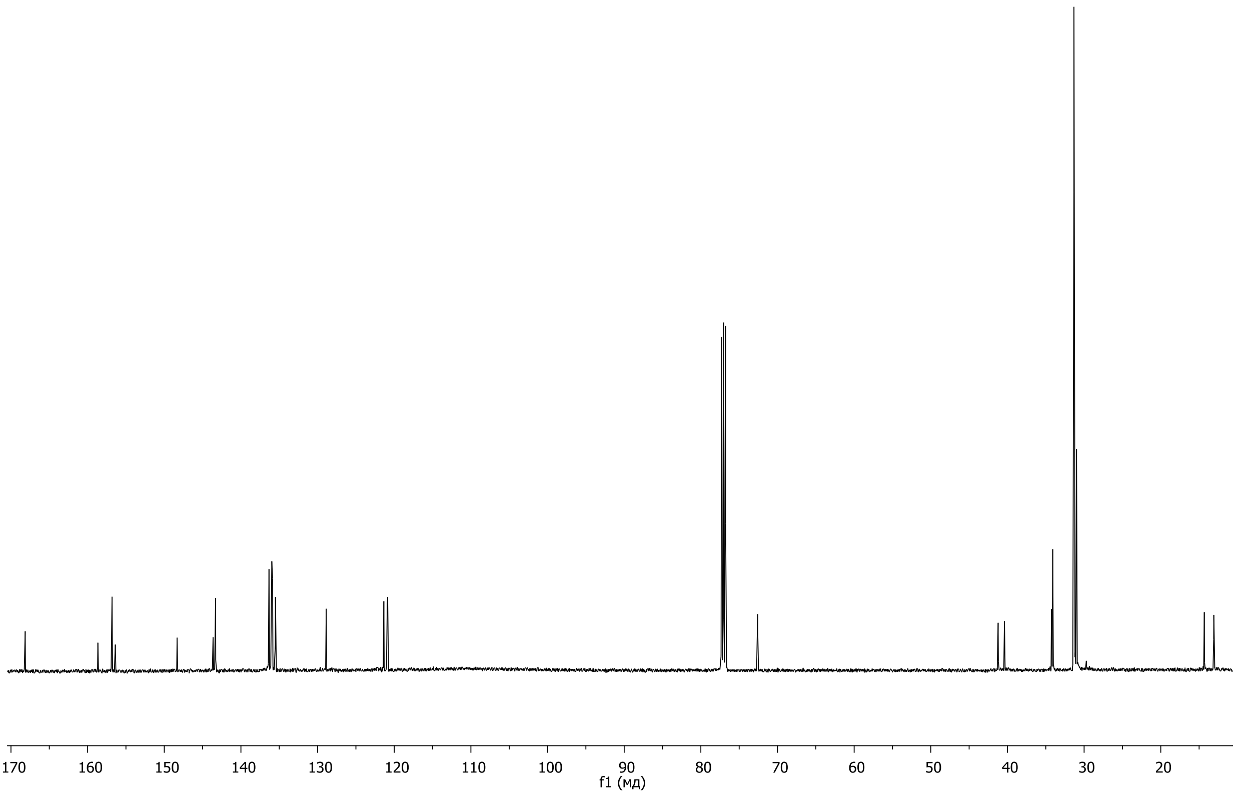

Figure S6: ^{13}C NMR spectrum of compound **4** ($CDCl_3$, 298 K, 125 MHz).

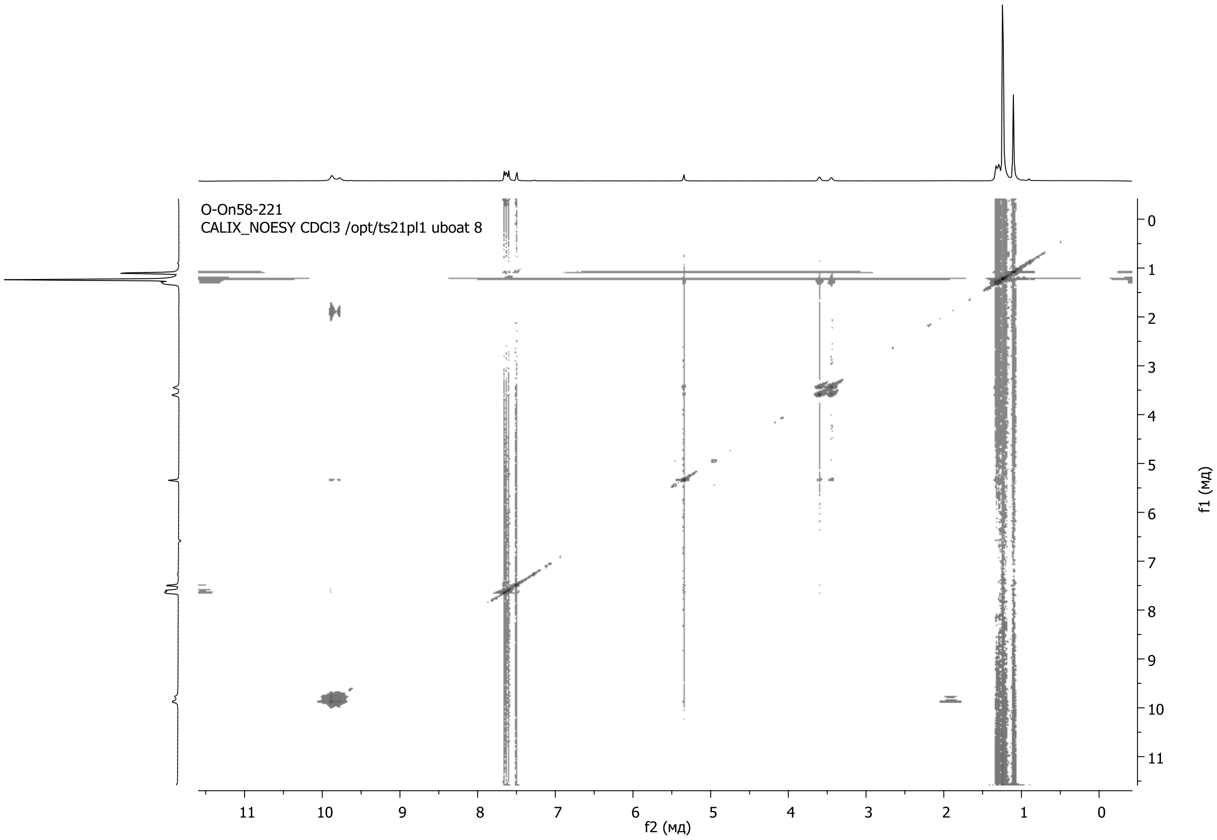

Figure S7: The 2D NMR ^1H-^1H NOESY spectrum of compound **4** ($CDCl_3$, 298 K, 500 MHz).

The peaks for the molecular ion (m/z ($M+H^+$) = 834.7) and the macrocycle with sodium (m/z ($M+Na^+$) = 856.8) and potassium (m/z ($M+K^+$) = 872.6) cations are observed in the MALDI-TOF mass spectrum of the compound **4** (M ($C_{46}H_{59}NO_5S_4$) = 833.3).

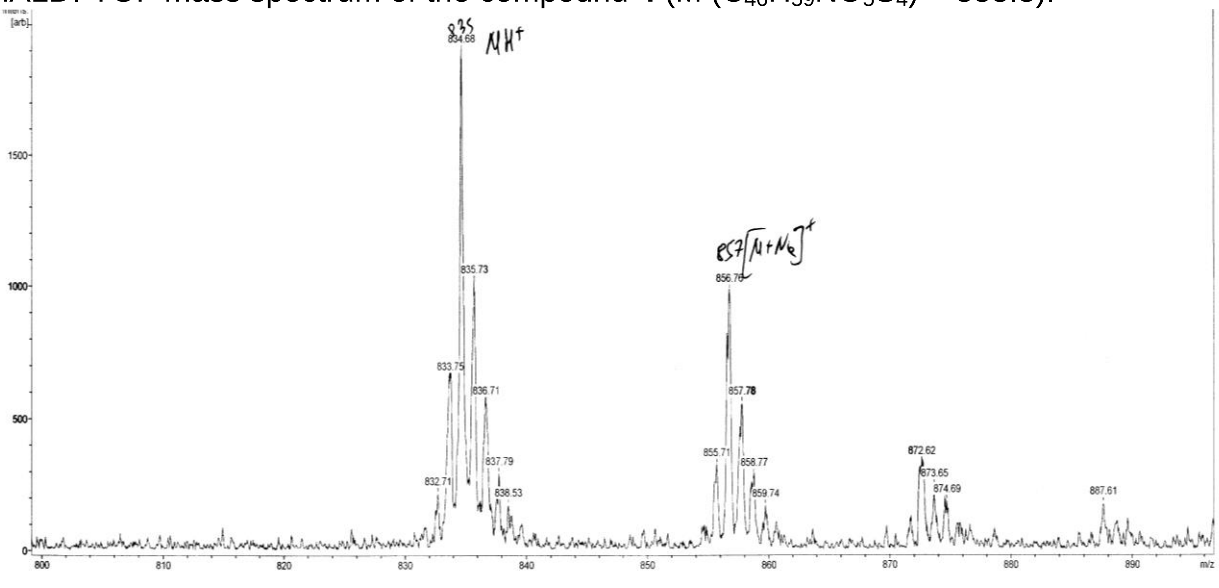

Figure S8: MALDI–TOF mass spectrum of compound **4**.

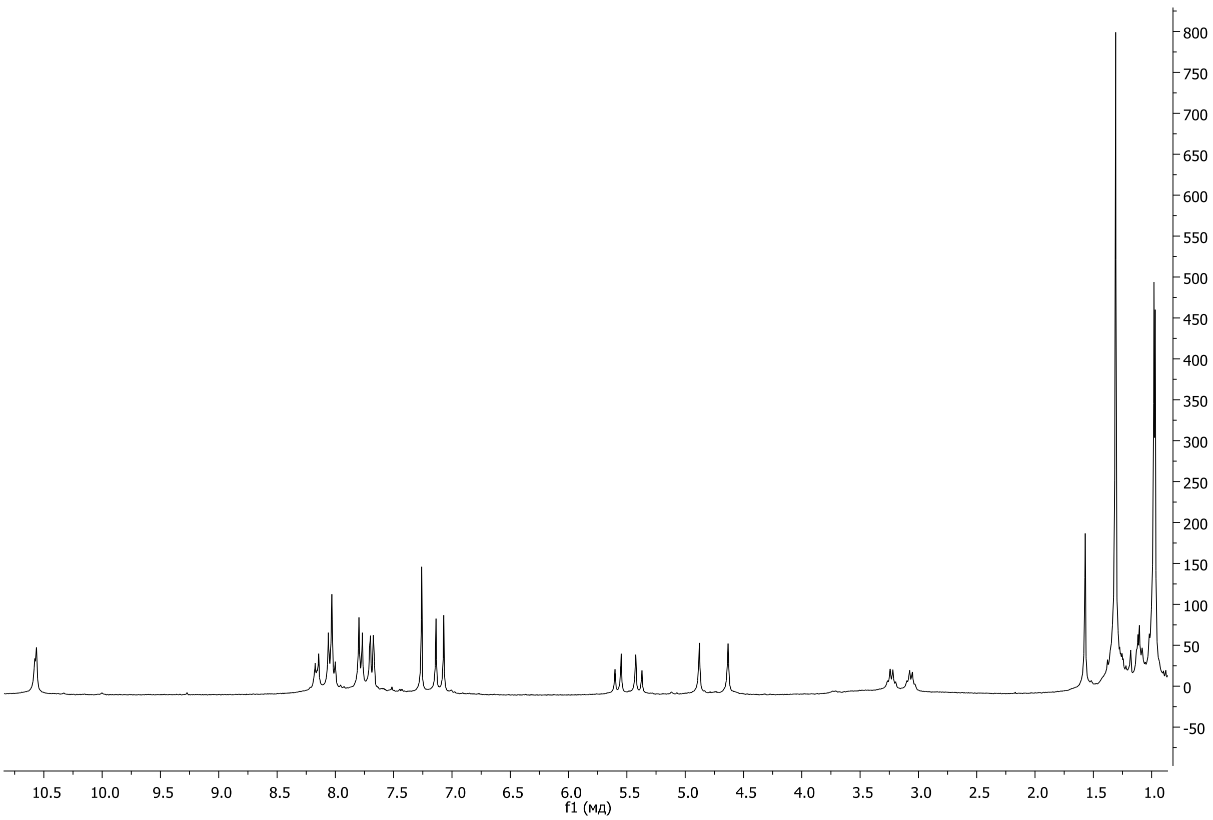

Figure S9: ^{1}H NMR spectrum of compound **5** ($CDCl_3$, 298 K, 300 MHz).

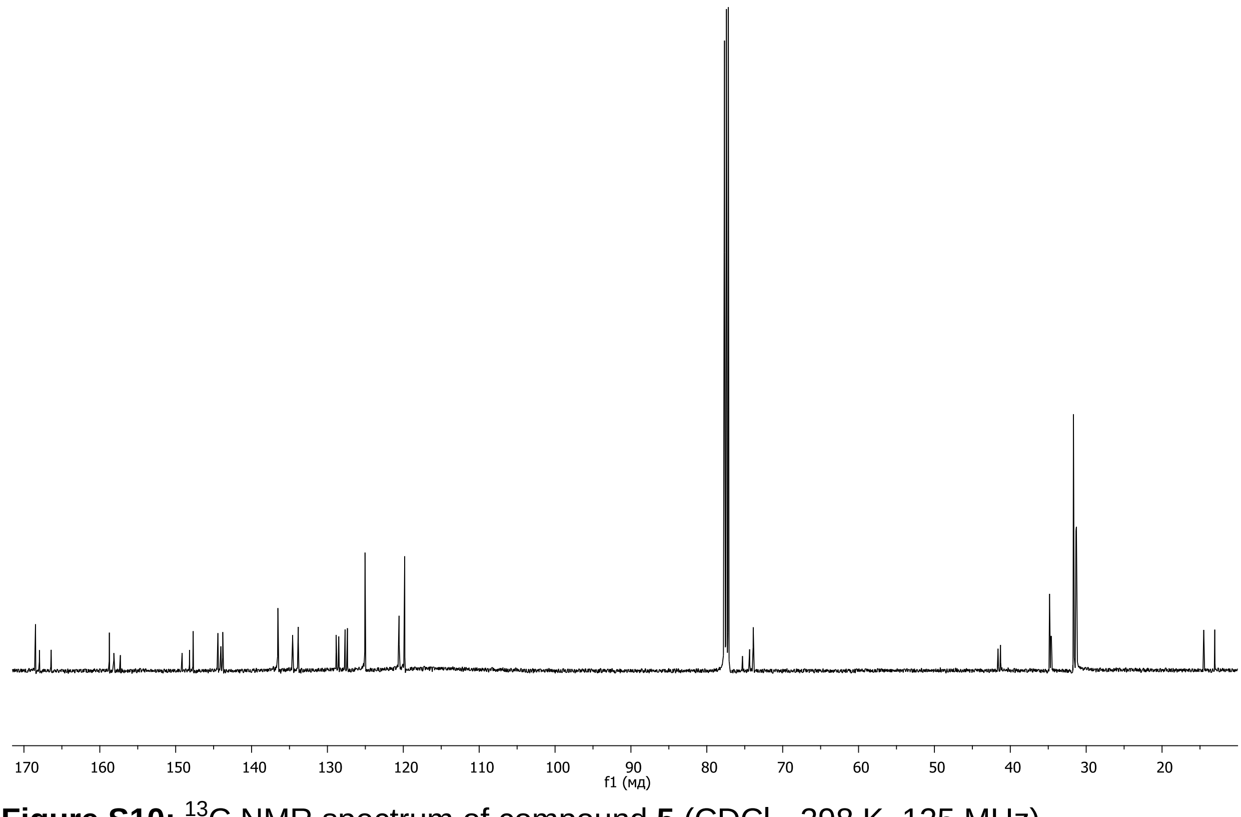

Figure S10: ^{13}C NMR spectrum of compound **5** ($CDCl_3$, 298 K, 125 MHz).

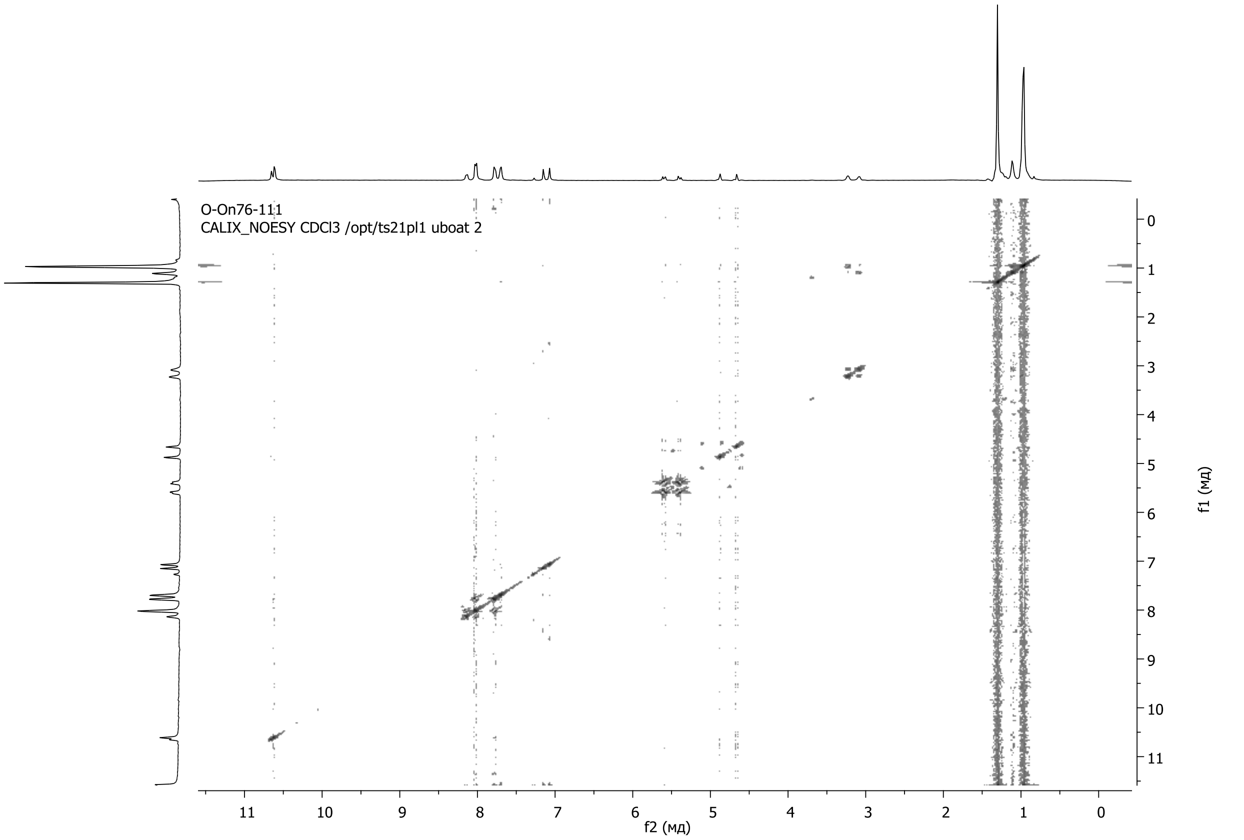

Figure S11: The 2D NMR ^{1}H-^{1}H NOESY spectrum of compound **5** ($CDCl_3$, 298 K, 500 MHz).

In the MALDI–TOF mass spectrum of the compound **5** (M ($C_{70}H_{77}N_7O_{14}S_4$) = 1367.4), a peak for the molecular ion with sodium cation (m/z ($M+Na^+$) = 1390.6) is observed.

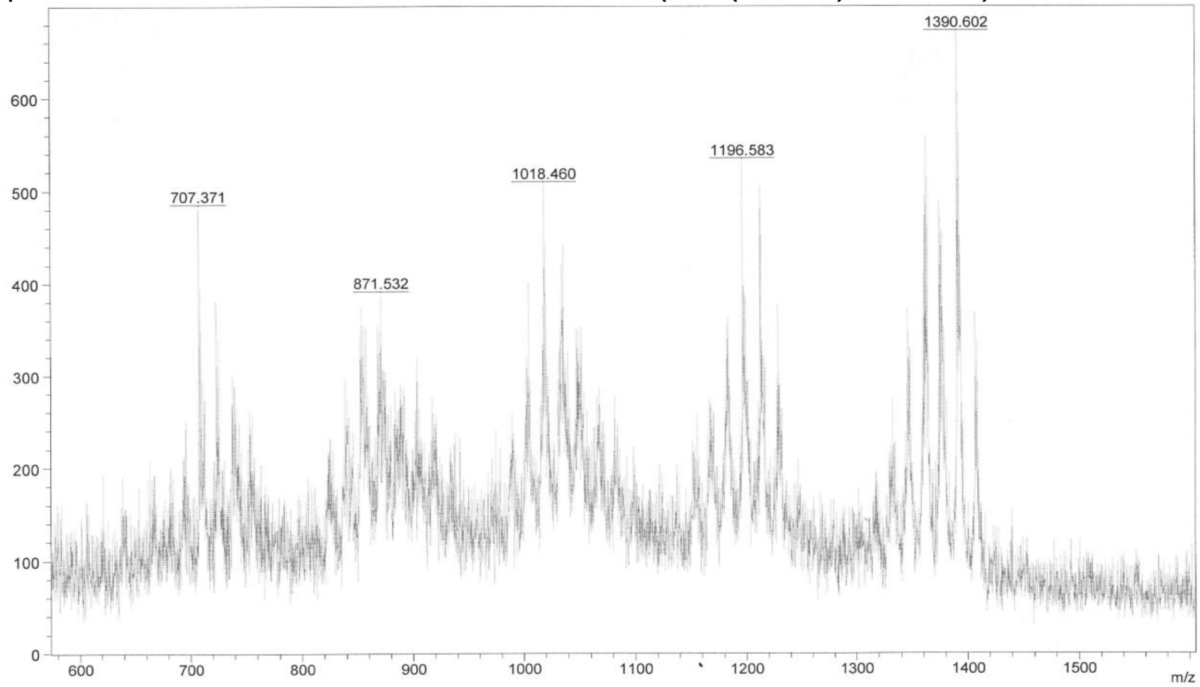

Figure S12: MALDI–TOF mass spectrum of compound **5**.

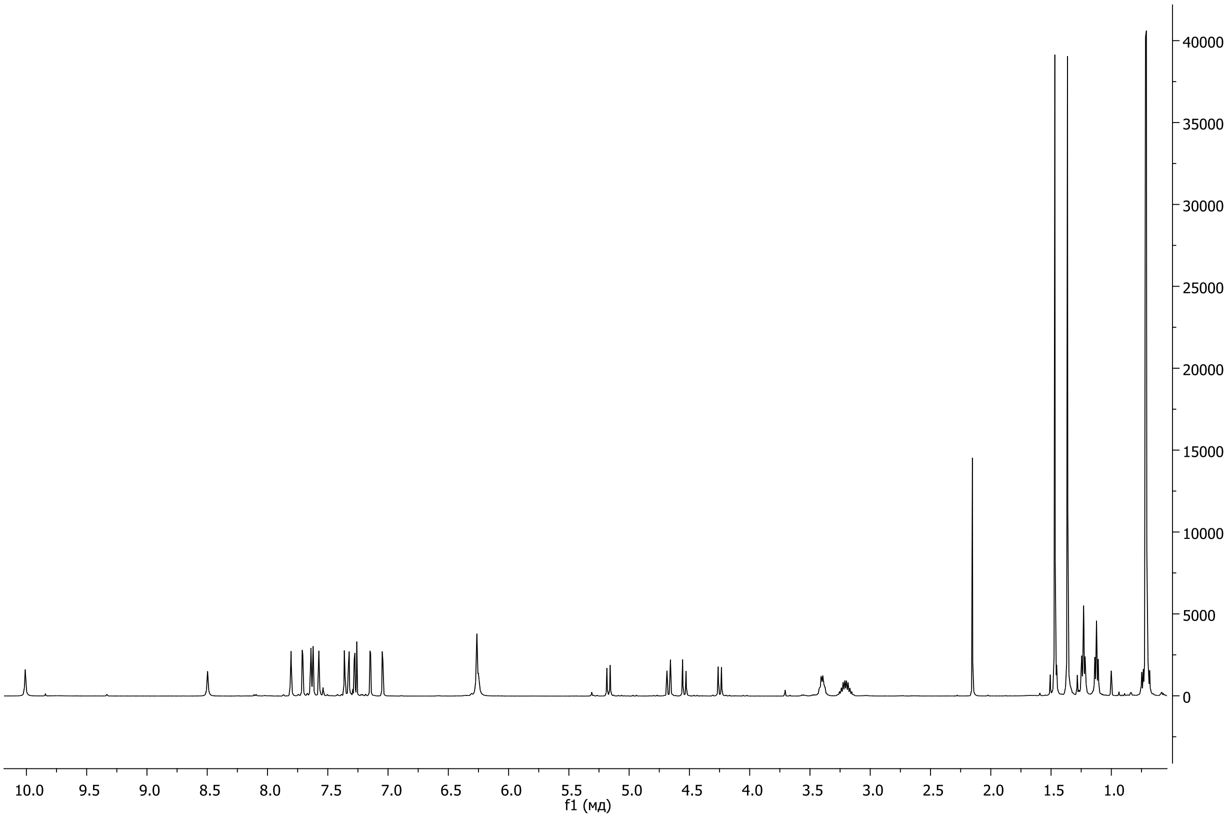

Figure S13: ^{1}H NMR spectrum of compound **6** ($CDCl_3$, 298 K, 300 MHz).

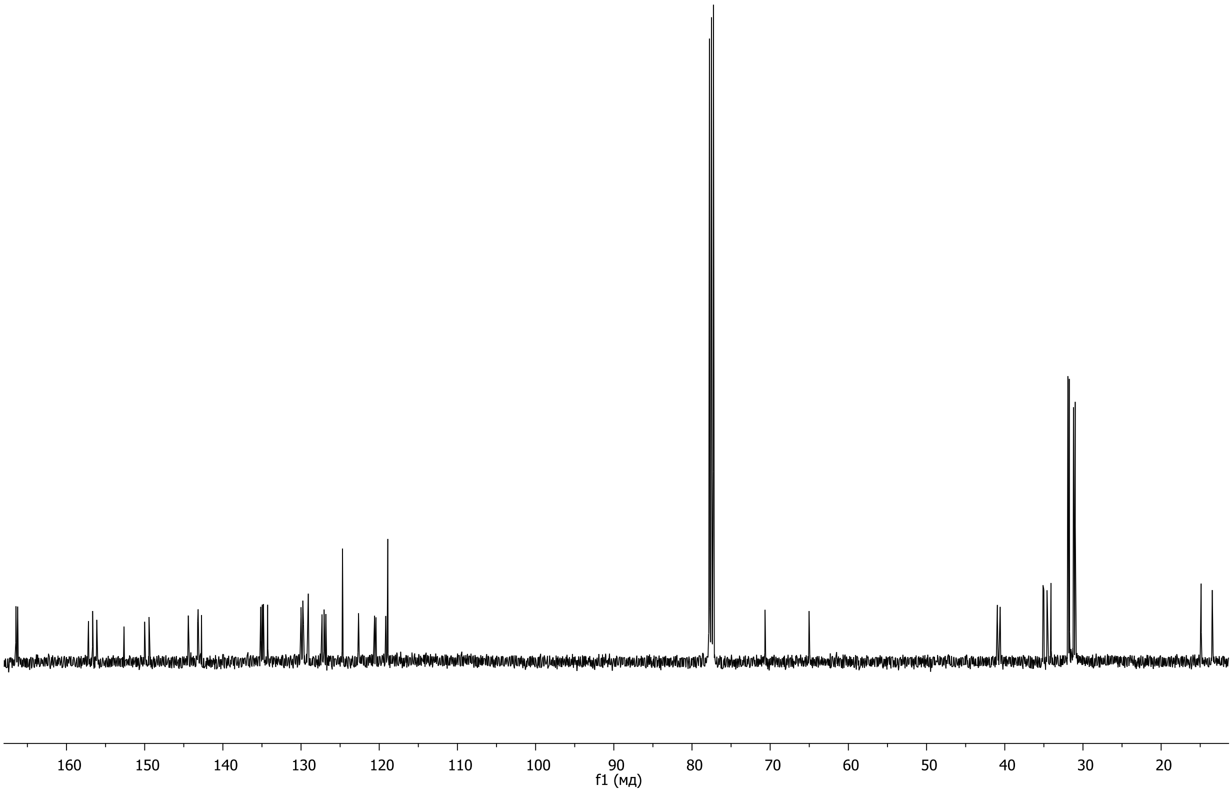

Figure S14: ^{13}C NMR spectrum of compound **6** ($CDCl_3$, 298 K, 125 MHz).

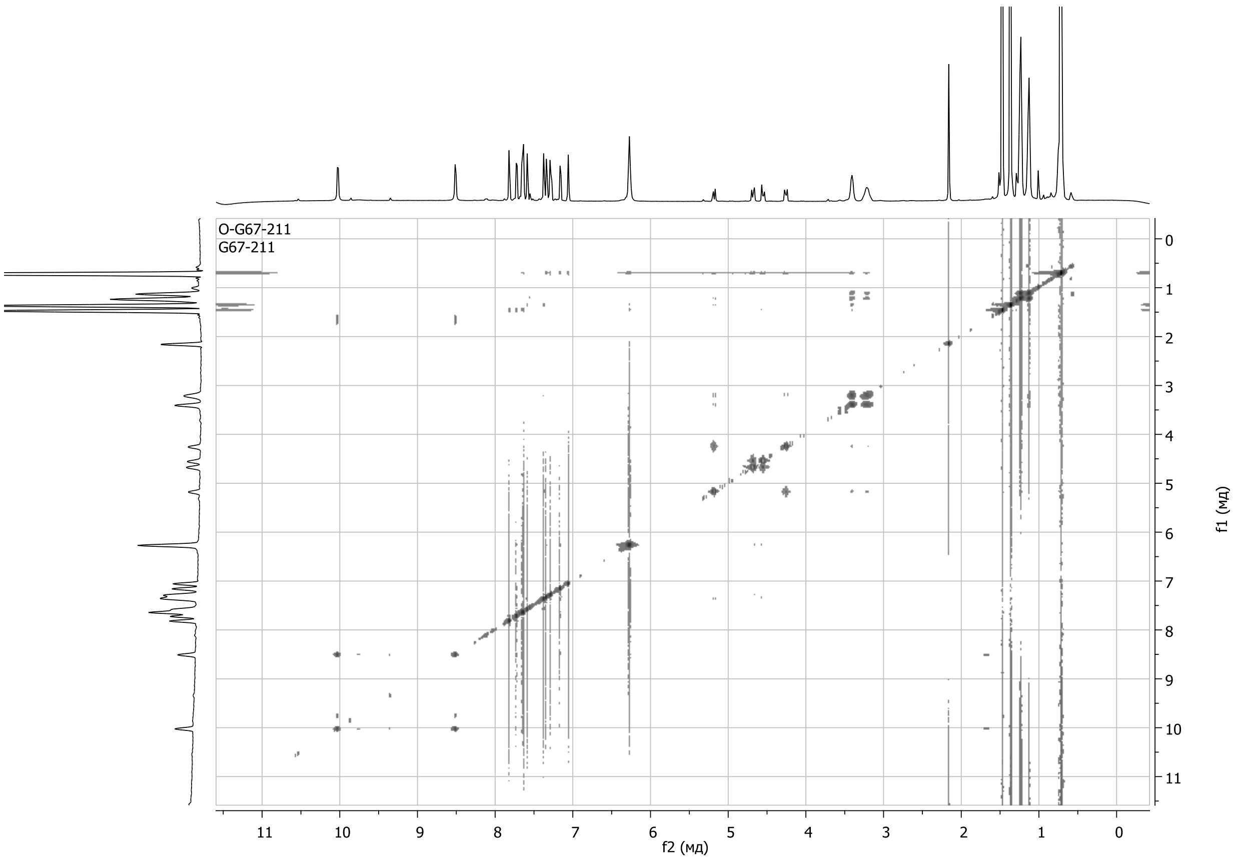

Figure S15: The 2D NMR ^{1}H-^{1}H NOESY spectrum of compound **6** ($CDCl_3$, 298 K, 500 MHz).

In the MALDI–TOF mass spectrum of compound **6** (M ($C_{54}H_{65}N_3O_8S_4$) = 1011.4), the peaks of molecular ion with sodium (m/z ($M+Na^+$) = 1034.5) and potassium (m/z ($M+K^+$) = 1050.5) cations are observed.

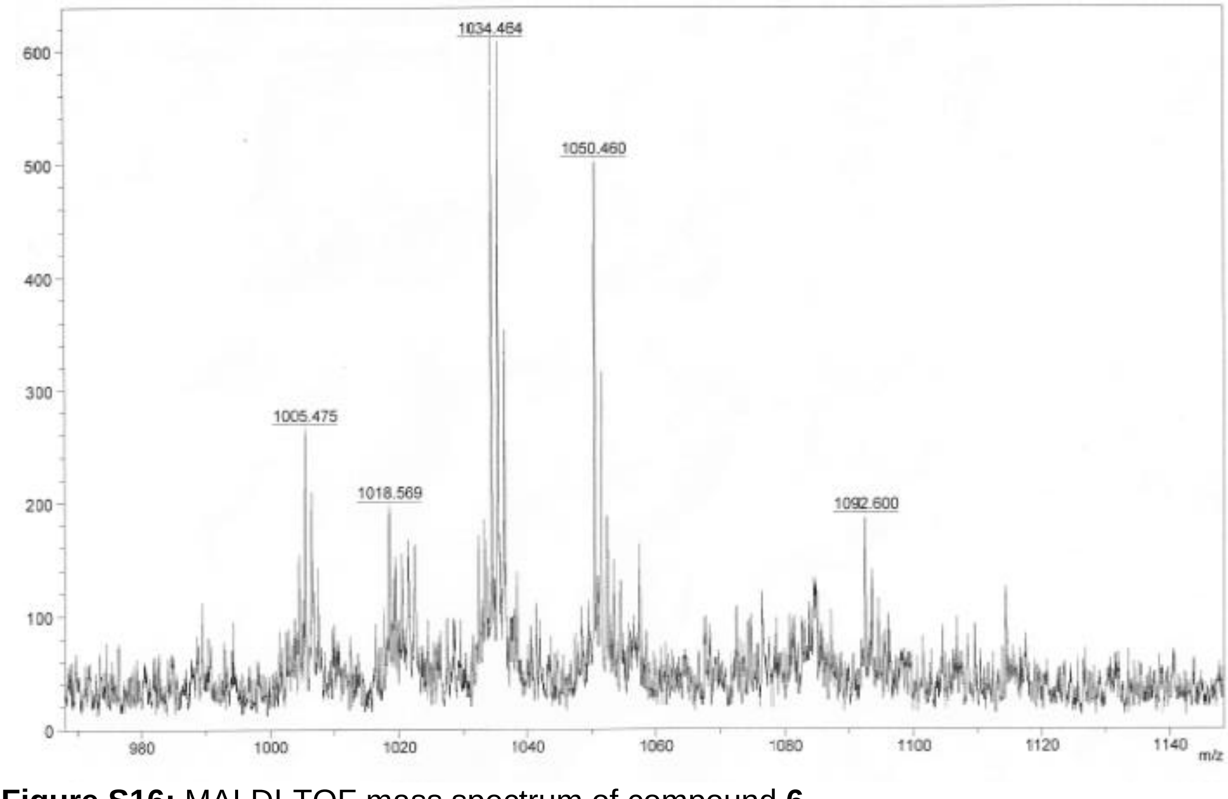

Figure S16: MALDI-TOF mass spectrum of compound **6**.

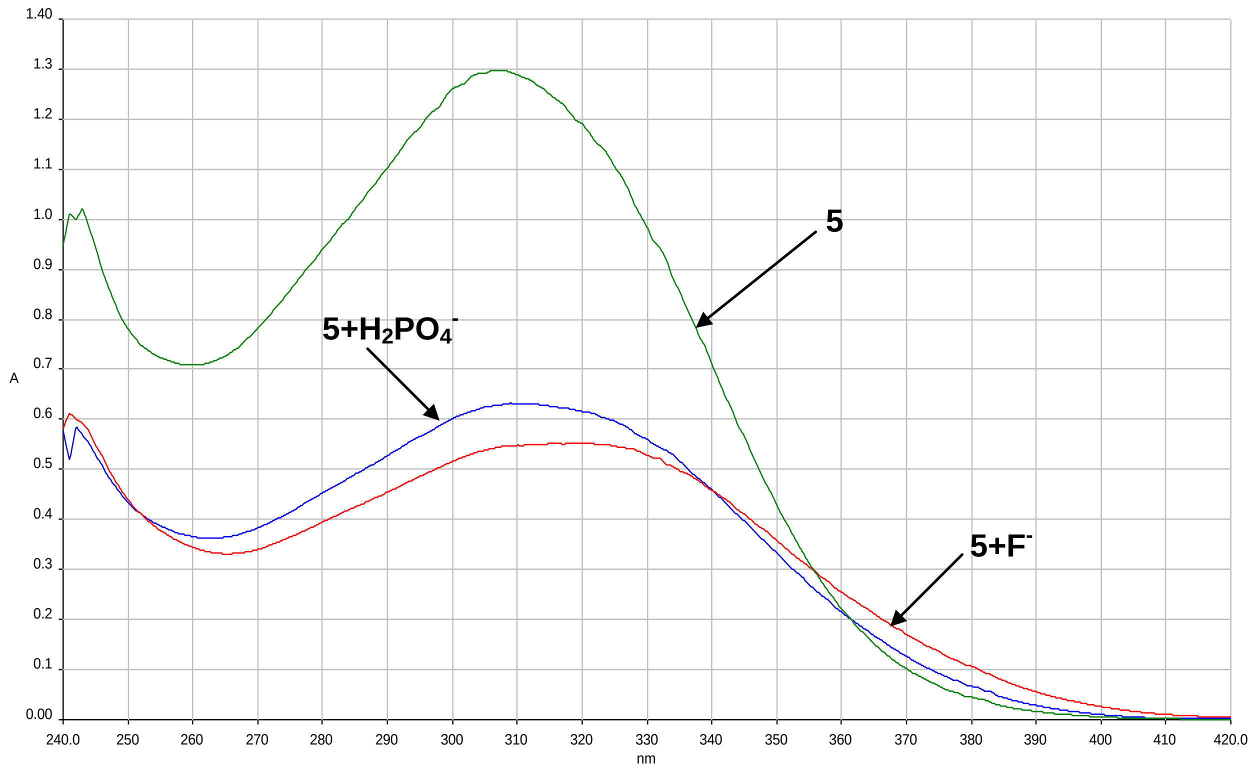

Figure S17: Changes in the absorption spectra of compound **5** after adding tetrabutylammonium salts ($CHCl_3$, $c_{n\text{-Bu4NX}}/c(\mathbf{5}) = 200$).

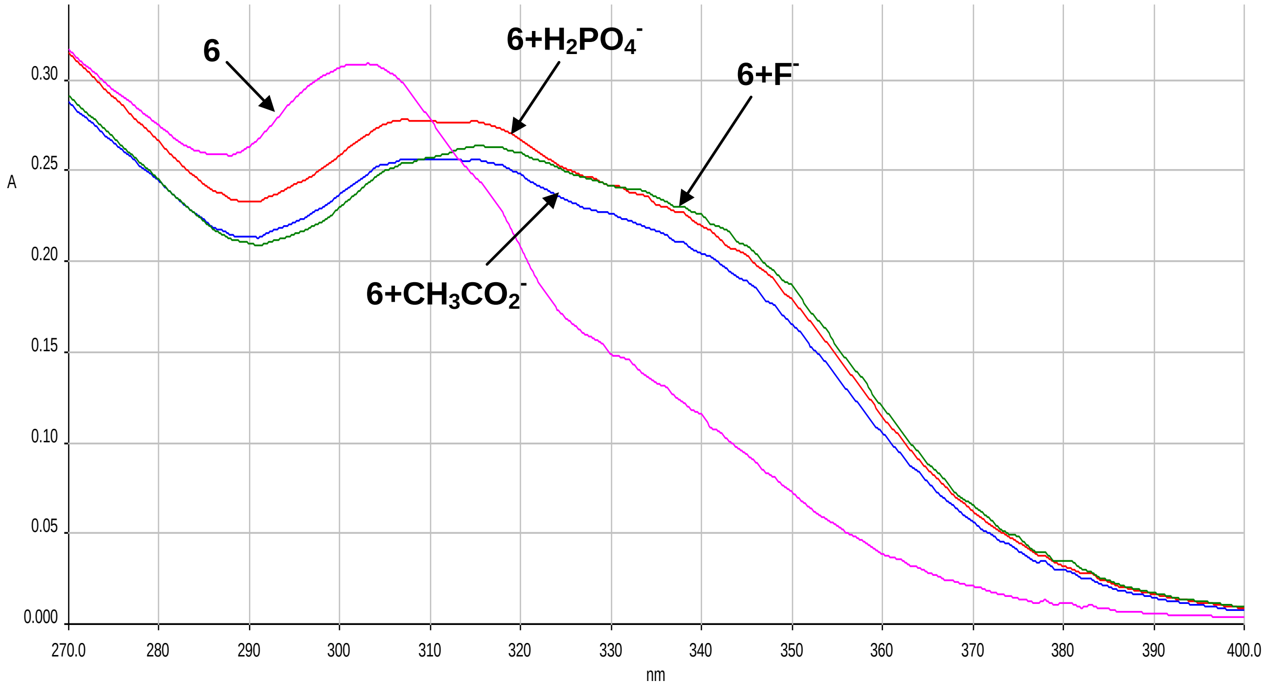

Figure S18: Changes in the absorption spectra of compound **6** after adding tetrabutylammonium salts ($CHCl_3$, $c_{n\text{-Bu4NX}}/c(\mathbf{6}) = 200$).

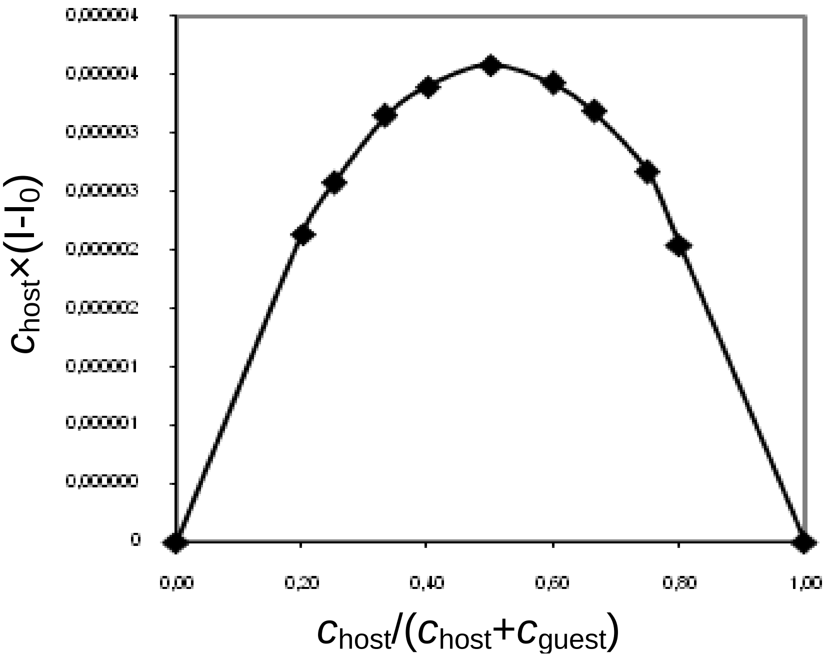

Figure S19: Job's plot for the **6**+*n*-$Bu_4NH_2PO_4$ system.

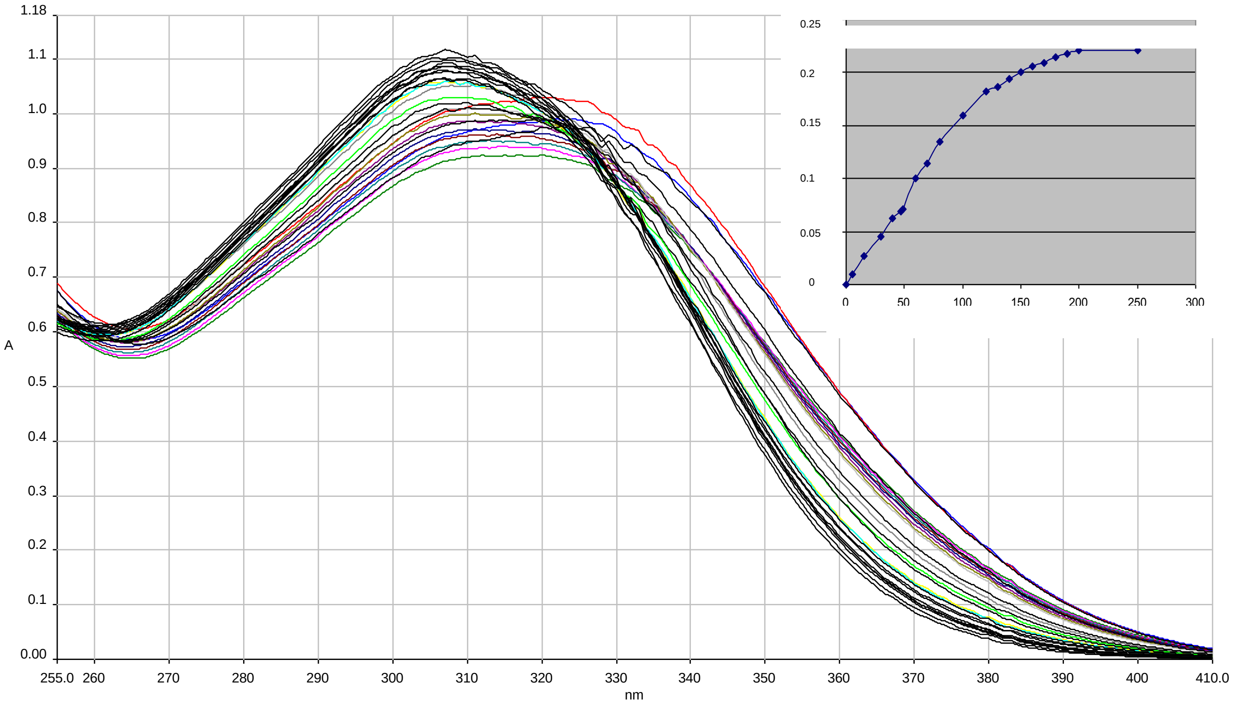

Figure S20: UV absorbtion spectra of the complexation system obtained by titration of the receptor **5** and fluoride ions. Inset: titration curve (c(**5**) = 2.5×10^{-5} M).

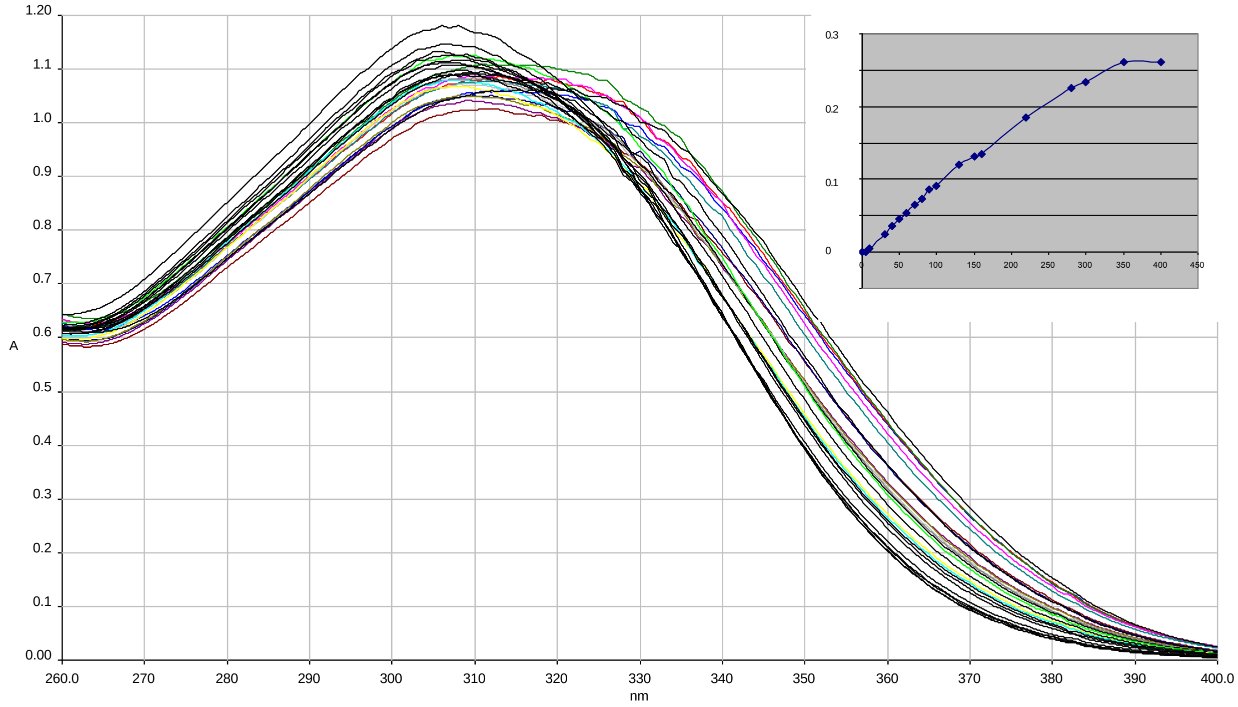

Figure S21: UV absorbtion spectra of the complexation system obtained by titration of the receptor **5** and dihydrogen phosphate ion. Inset: titration curve (c(**5**) = 2.5 × 10^{-5} M).

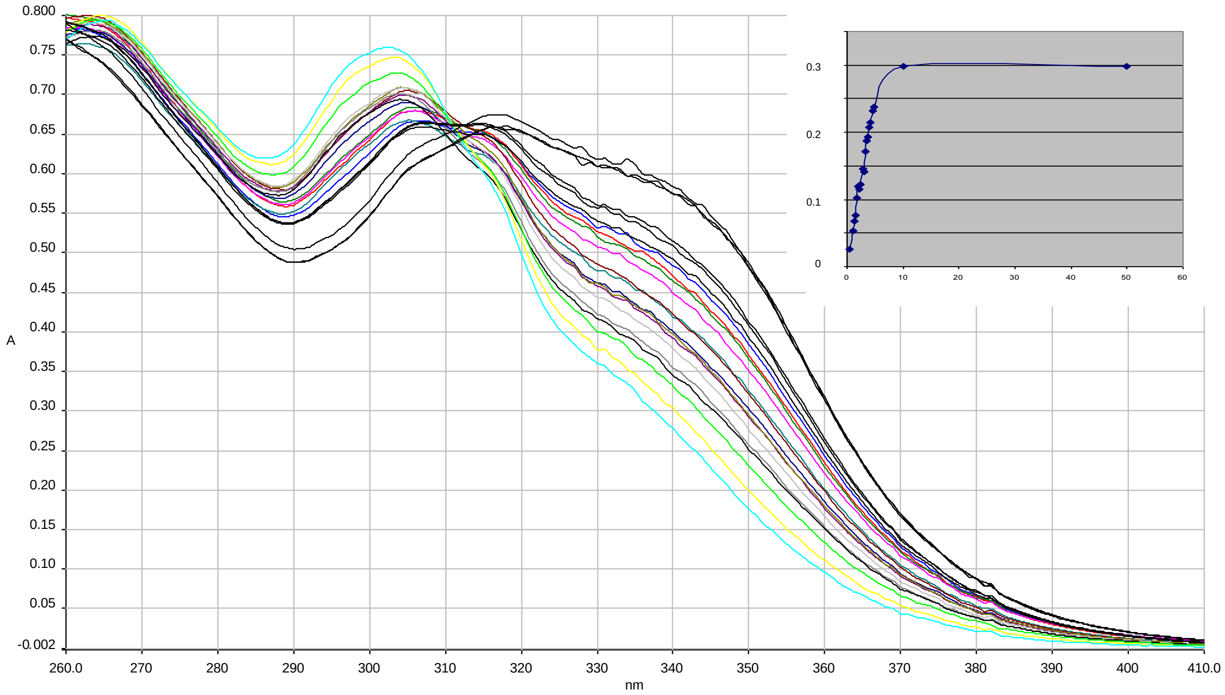

Figure S22: UV absorbtion spectra of the complexation system obtained by titration of the receptor **6** and fluoride ion. Inset: titration curve (c(**6**) = 2.5 × 10^{-5} M).

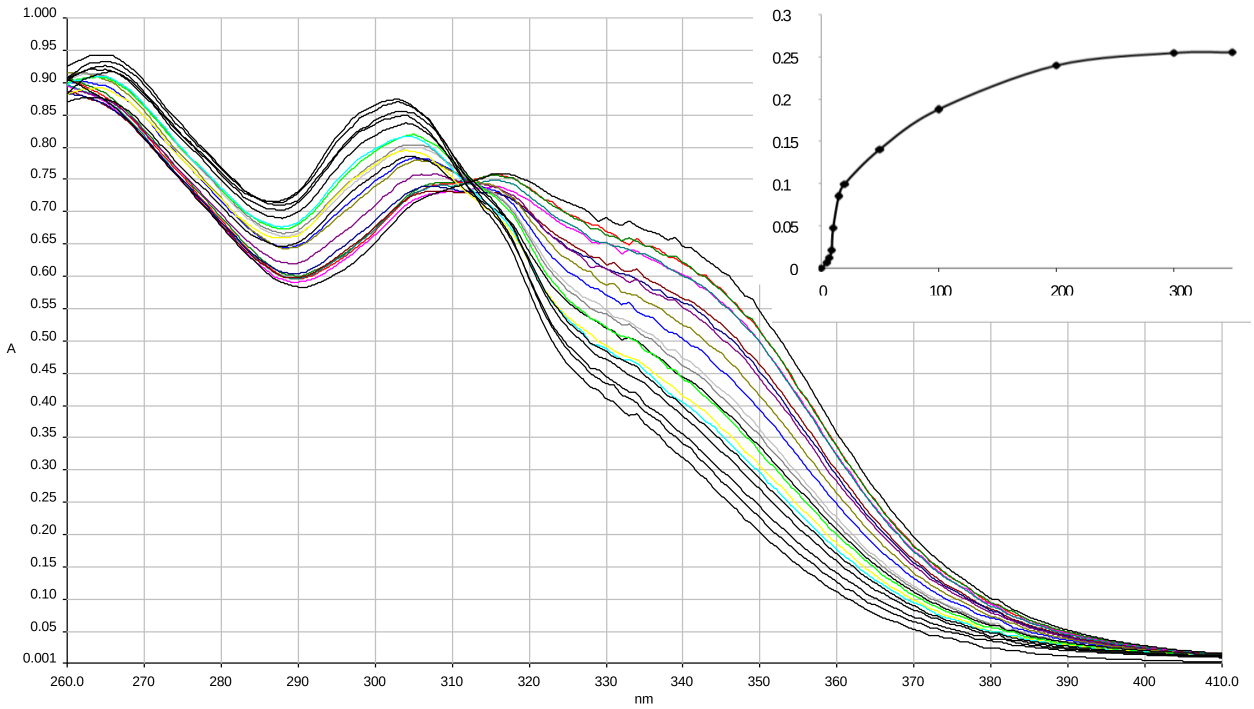

Figure S23: UV absorbtion spectra of the complexation system obtained by titration of the receptor **6** and dihydrogen phosphate ion. Inset: titration curve (c(**6**) = 2.5 × 10^{-5} M).

References

1. Stoikov, I. I.; Ibragimova, D. S.; Shestakova, N. V.; Krivolapov, D. B.; Litvinov, I. A.; Antipin, I. S.; Konovalov, A. I.; Zharov, I. *Supramolecular Chemistry* **2009**, *21*, 564-571.

2. Stoikov, I. I.; Yantemirova, A. A.; Nosov, R. V.; Rizvanov, I. Kh.; Julmetov, A. R.; Klochkov, V. V.; Antipin, I. S.; Konovalov, A. I.; Zharov, I. *Org. Biomol. Chem*. **2011**, *9*, 3225-3234.

3. Hirose, K. *J. Incl. Phenom. Macrocycl. Chem.* **2001**, *39*, 193–209.
